# Integrated Assessment of Neurobehavioral and Cardiotoxic Effects of Pyrrolidine-Containing Cathinones in Zebrafish: Structural Determinants of Functional Safety Profiles

**DOI:** 10.3390/ijms27073141

**Published:** 2026-03-30

**Authors:** Ouwais Aljabasini, Niki Tagkalidou, Martalu D. Pazos, Guillermo García-Díez, Eva Prats, Roger Seco, Xavier Berzosa, Raúl López-Arnau, Demetrio Raldua

**Affiliations:** 1 Institute for Environmental Assessment and Water Research (IDAEA-CSIC), Jordi Girona, 18, 08034 Barcelona, Spain; ouwais.aljabasini@idaea.csic.es (O.A.); niki.tagkalidou@idaea.csic.es (N.T.); email@rogerseco.cat (R.S.); 2 Department of Pharmacology, Toxicology and Therapeutic Chemistry, Faculty of Pharmacy and Food Sciences, Pharmacology Section, Institute of Biomedicine (IBUB), University of Barcelona, 08028 Barcelona, Spain; martaludominguez@ub.edu (M.D.P.); raullopezarnau@ub.edu (R.L.-A.); 3 Chemical Reactions for Innovative Solutions (CRISOL), IQS School of Engineering, Universitat Ramon Llull, 08017 Barcelona, Spain; guillermo.garcia@iqs.url.edu (G.G.-D.); xavier.berzosa@iqs.url.edu (X.B.); 4 Research and Development Center (CID-CSIC), Jordi Girona, 18, 08034 Barcelona, Spain; epmbmc@cid.csic.es

**Keywords:** synthetic cathinones, new psychoactive substances (NPS), zebrafish embryo, cardiotoxicity, atrioventricular block, structure–activity relationship (SAR), new approach methodologies (NAMs)

## Abstract

The rapid emergence of New Psychoactive Substances (NPS), particularly pyrrolidinophenone derivatives, poses a significant challenge for public health and forensic toxicology. While their neuropharmacological profiles as dopamine transporter inhibitors are well-documented, their cardiac toxicity remains poorly understood. This study employs a multiparametric New Approach Methodology (NAM) using zebrafish embryos to integrate neurobehavioral and cardiotoxic endpoints for comparative hazard prioritization. We evaluated nine pyrrolidine-containing cathinones, including α-PVP, MDPV, α-PiHP, MDPiHP, α-D2PV, 3-Cl-, 4-Cl-, and 3,4-Cl-α-PVP, and 4-F-3-Me-α-PVP, on locomotor activity and cardiac rhythmicity using high-speed video microscopy and dynamic pixel analysis. Across the series, compounds induced concentration-dependent negative chronotropy and, in most cases, locomotor suppression. Crucially, we identified a functional dissociation between atrial rate control and atrioventricular (AV) conduction. The 3,4-dichloro substitution (3,4-Cl-α-PVP) was the most potent inducer of negative chronotropy (EC_50_ = 52.6 μM), whereas 4-Cl-α-PVP exhibited a distinct pro-arrhythmic liability, increasing the incidence of 2:1 AV block. Time-course locomotor profiling indicated that α-PVP and chlorinated analogs were among the most potent behavioral modifiers. Using a Functional Safety Index (AV block EC_50_/locomotor EC_50_-like), we show that most compounds exhibit wide separations between neurobehavioral inhibition and severe conduction impairment, while specific substitutions, particularly para-chlorination, are associated with comparatively reduced functional separation between these endpoints within the assay. Overall, these data demonstrate that subtle structural changes within the pyrrolidinophenone scaffold can shape distinct arrhythmic phenotypes and functional safety profiles, supporting zebrafish-based integrated screening as a rapid platform for prioritizing emerging synthetic cathinones with comparatively higher cardiac liability within this experimental framework.

## 1. Introduction

New psychoactive substances (NPS) are synthetic drugs designed to reproduce the effects of controlled substances while circumventing existing legal restrictions [1]. Between 2013 and 2024, the annual number of newly identified NPS remained consistently high, reaching a record 688 in 2024 [2]. Their legal ambiguity, combined with frequent mislabeling and adulteration, leaves users unaware of the exact substances they consume and increases the likelihood of unintended exposure [3,4]. Consistently, postmortem data indicate that polydrug patterns are common among NPS-related fatalities [5].

Synthetic cathinones constitute a major subgroup of NPS and have become increasingly prominent within the European stimulant market [6,7]. These compounds are structural analogues of cathinone, the psychoactive alkaloid of *Catha edulis*, and their primary pharmacology often involves inhibition of monoamine transporters, with potency and transporter selectivity shaped by side-chain and aromatic substitutions [8,9]. In particular, pyrrolidinophenone (pyrovalerone-related) cathinones are typically strong dopamine (DA) and norepinephrine (NE) transporter (DAT and NET, respectively) inhibitors [10,11] and have been repeatedly implicated in acute intoxications [5,12,13].

Beyond their psychostimulant, rewarding and reinforcing effects [8,14], and despite the extensive characterization of the neuropharmacological profiles of pyrrolidinophenones as potent, non-substrate DAT inhibitors [11,15], their systemic toxicity remains critically understudied. This lack of experimental data stands in stark contrast to clinical and forensic reports, which frequently link synthetic cathinone overdose to severe cardiovascular complications, including tachycardia, hypertension, and fatal arrhythmias [16,17]. However, it remains unclear whether the structural modifications that enhance neurobehavioral potency also increase cardiac liability, or whether these two effect domains can become dissociated across closely related analogues. Moreover, while hyperthermia is known to exacerbate cardiac risk in homeothermic mammals, the direct cardiac liabilities and structure-dependent differences across closely related cathinones remain insufficiently characterized, limiting evidence-based risk assessment for newly emerging analogues [4,18].

To address this gap, New Approach Methodologies (NAMs) are needed to provide multiparametric, time-resolved functional data that traditional rodent models often lack due to throughput constraints and physiological differences. The zebrafish (*Danio rerio*) embryo has emerged as a powerful platform for integrated toxicity assessments, offering high genomic homology to humans and a remarkably similar cardiac physiology [19]. Notably, the transparency of the eleutheroembryos allows for the non-invasive, real-time monitoring of both neurobehavioral endpoints (locomotor activity) and cardiac performance (atrial and ventricular rhythmicity), providing a common in vivo platform for comparative assessment of central and peripheral effects.

**Figure 1:**
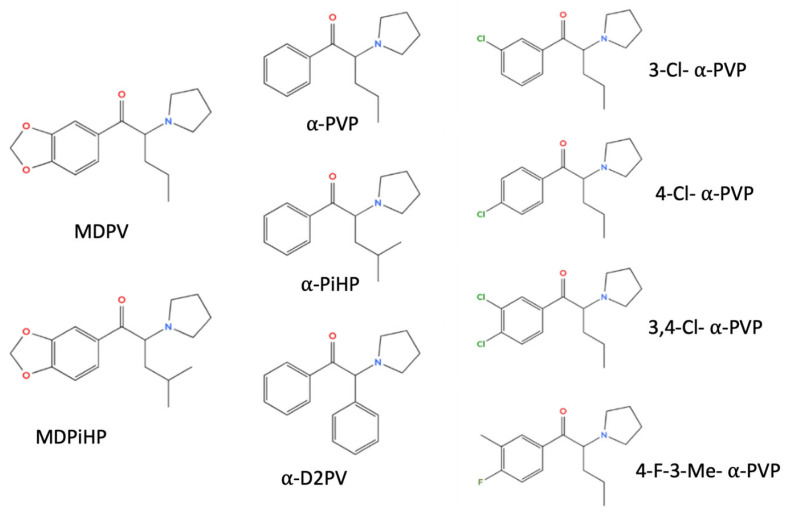
Chemical structure of α-PiHP, α-PVP, MDPiHP, MDPV, α-D2PV, 3-Cl-α-PVP, 4-Cl-α-PVP, 3,4-Cl-α-PVP, 4-F-3-Me-α-PVP. Oxygen atoms are shown in red, nitrogen atoms in blue, and halogen substituents in green for clarity.

In the present study, we tested the hypothesis that structural modifications within the pyrrolidinophenone scaffold differentially influence neurobehavioral potency and cardiac liability, and that these effects may remain coupled in some analogues but become functionally dissociated in others. To address this, we selected a panel of nine pyrrolidine-containing cathinones representing relevant structural variation across the scaffold, including parent compounds, chlorinated analogues, methylenedioxy derivatives, and branched side-chain variants. These included established compounds such as α-pyrrolidinopentiophenone (α-PVP) and 3,4-methylenedioxy-α-pyrovalerone (MDPV), as well as more recently emerged derivatives such as α-pyrrolidinoisohexanophenone (α-PiHP), 3,4-methylenedioxy-α-pyrrolidinoisohexanophenone (MDPiHP), α-pyrrolidino-2-phenylacetophenone (α-D2PV), 3-chloro-, 4-chloro-, and 3,4-dichloro-α-pyrrolidinovalerophenone (3-Cl-, 4-Cl-, and 3,4-Cl-α-PVP, respectively), and 4-fluoro-3-methyl-α-pyrrolidinovalerophenone (4-F-3-Me-α-PVP) (Figure 1). By integrating time-resolved locomotor profiling with dynamic pixel-based analysis of cardiac rhythmicity and conduction, we aimed to compare the degree of functional separation between neurobehavioral effects and cardiotoxic endpoints across this series. This multiparametric approach provides a framework for comparative hazard prioritization of newly emerging synthetic cathinones.

## 2. Results

### 2.1. Effects of Pyrrolidinophenone Derivatives on Atrial Chronotropy

The effects of the tested pyrrolidinophenone derivatives on atrial chronotropy were quantified as percentage inhibition of atrial rate relative to individual control values. Concentration–response relationships were modeled using a four-parameter logistic function constrained between 0 and 100%, fitted to individual replicate data at concentrations ≥ 5 µM. Parameter uncertainty was assessed by nonparametric bootstrap resampling (*n* = 2000), stratified by concentration.

**Figure 2:**
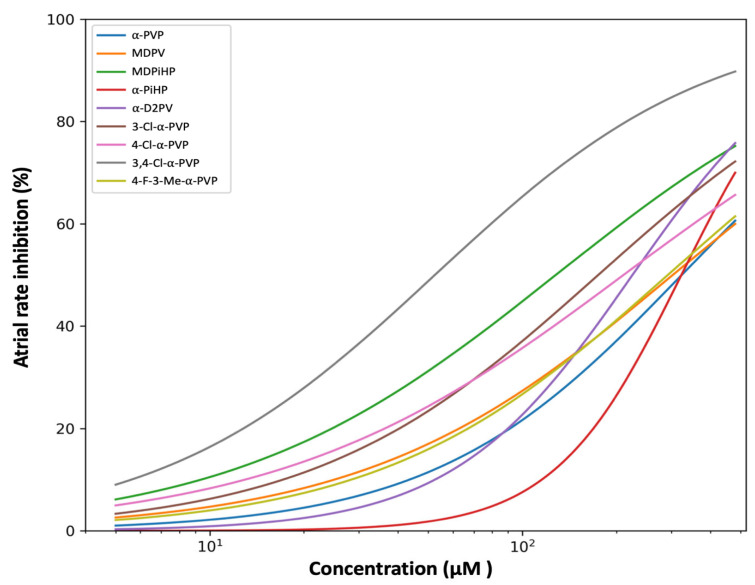
Effects of pyrrolidinophenone derivatives on atrial chronotropy in zebrafish embryos. Comparative concentration–response curves showing atrial rate inhibition induced by the indicated pyrrolidinophenone derivatives in zebrafish embryos. Atrial chronotropy is expressed as the percentage inhibition of atrial rate relative to individual control values. Curves represent four-parameter logistic (4PL) model fits constrained between 0 and 100%, fitted to individual replicate data at concentrations ≥ 5 µM. Shaded areas indicate 95% confidence intervals derived from nonparametric bootstrap resampling (*n* = 2000). Concentration is shown on a logarithmic scale. Confidence intervals reflect model uncertainty and should be considered when comparing apparent potency differences across compounds.

All compounds produced concentration-dependent negative chronotropic effects, although marked differences in potency and curve shape were observed across the series (Figure 2 and Supplementary Figure S2). When modeled using replicate-level data, atrial chronotropic potency spanned more than a six-fold range across compounds.

Model-derived EC_50_ and Hill slope estimates for all compounds are summarized in Table 1. The unsubstituted compound α-PVP displayed a moderate atrial chronotropic effect (EC_50_ = 323.9 µM, 95% CI: 284.3–375.0 µM) with a shallow Hill slope, indicating a gradual and heterogeneous response. Among the mono-chlorinated analogues, 3-Cl-α-PVP (EC_50_ = 175.2 µM) was more potent than 4-Cl-α-PVP (EC_50_ = 211.1 µM).

Notably, the di-chlorinated analogue 3,4-Cl-α-PVP exhibited the strongest atrial chronotropic effect in the series (EC_50_ = 52.6 µM), producing substantial atrial rate inhibition already at low concentrations. Side-chain modifications also modulated atrial chronotropy, with MDPiHP (EC_50_ = 128.2 µM) markedly more potent than MDPV (EC_50_ = 303.4 µM), whereas 4-F-3-Me-α-PVP displayed comparatively weak chronotropic activity (EC_50_ = 292.5 µM).

### 2.2. Effects of Pyrrolidinophenone Derivatives on Atrioventricular Conduction

In parallel, the effects of the compounds on AV conduction were assessed by quantifying AV block as the percentage of embryos exhibiting impaired atrial–ventricular coupling. Concentration–response relationships were modeled using a four-parameter logistic (4PL) function and fitted to individual replicate data by nonlinear least-squares regression. Parameter uncertainty was evaluated by nonparametric bootstrap resampling (*n* = 2000), as described above. Model-derived EC_50_ and Hill slope estimates for all compounds are summarized in Table 2.

**Figure 3:**
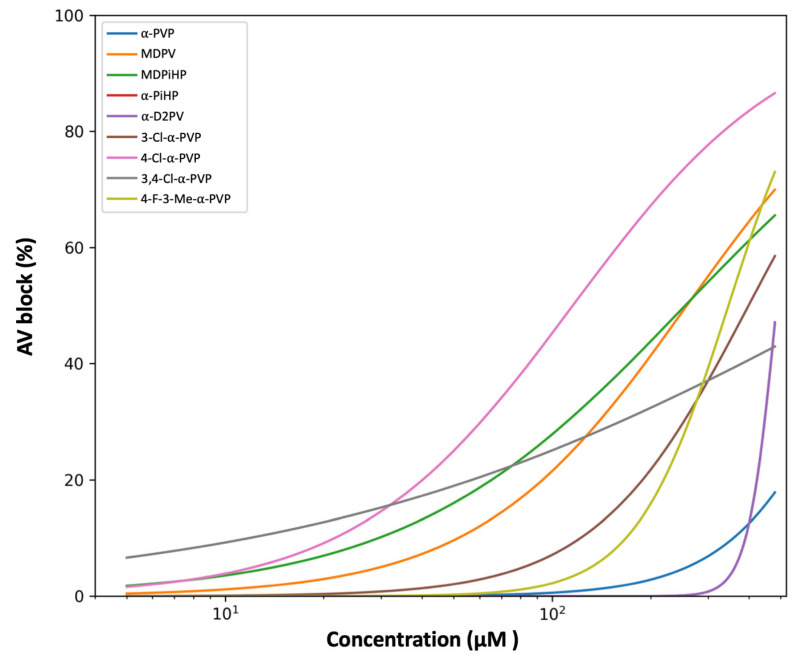
Effects of pyrrolidinophenone derivatives on atrioventricular conduction in zebrafish embryos. Comparative concentration–response curves showing the incidence of atrioventricular (AV) block induced by the indicated pyrrolidinophenone derivatives in zebrafish embryos. AV block is expressed as the percentage of embryos exhibiting impaired atrial–ventricular coupling. Curves represent four-parameter logistic (4PL) model fits constrained between 0 and 100%, fitted to replicate-level data at concentrations ≥5 µM. α-PiHP is included in the dataset but is not plotted because AV block incidence remained at 0% across most concentrations and increased only at the highest tested level, preventing stable curve fitting. Shaded areas indicate 95% confidence intervals derived from nonparametric bootstrap resampling (*n* = 2000). Concentration is shown on a logarithmic scale. Confidence intervals reflect model uncertainty and should be considered when comparing apparent potency differences across compounds.

In contrast to atrial chronotropy, AV-blocking potency varied widely across compounds and did not uniformly parallel atrial effects (Figure 3 and Supplementary Figure S3). When analyzed at concentrations ≥ 5 µM, 4-Cl-α-PVP showed the lowest point estimate for AV-blocking potency (EC_50_ = 115.8 µM), whereas α-PVP produced minimal AV block within the tested concentration range (EC_50_ = 937.0 µM).

MDPV and MDPiHP displayed comparable AV-blocking potencies, despite clear differences in their atrial chronotropic profiles. 4-F-3-Me-α-PVP exhibited intermediate AV-blocking activity, with concentration–response characteristics similar to those observed for atrial chronotropy.

Together, these results demonstrate that atrial chronotropy and AV conduction are differentially affected by structural modifications within the compound series, with several compounds displaying pronounced dissociation between the two functional endpoints.

### 2.3. Effects of Pyrrolidinophenone Derivatives on Locomotor Activity

**Figure 4:**
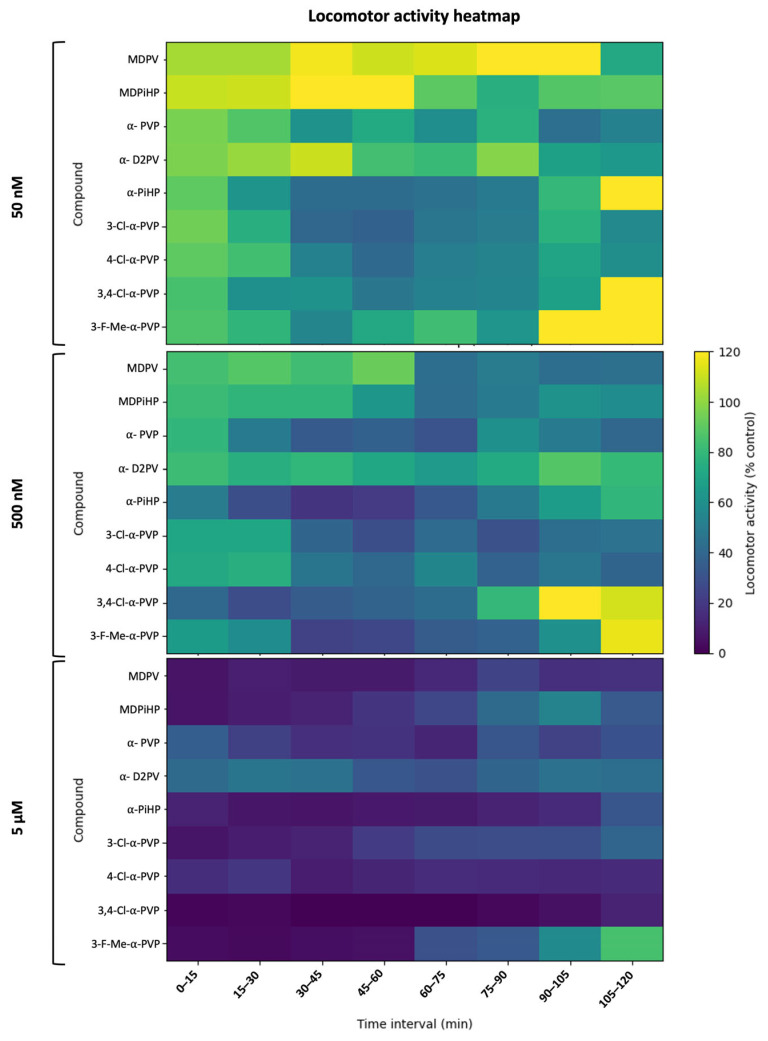
Kinetic heatmaps of locomotor activity under continuous exposure. Median normalized locomotor activity of 5 dpf zebrafish eleutheroembryos exposed continuously to each compound at 50 nM (top), 500 nM (middle), or 5 µM (bottom) over 120 min. Activity was quantified in consecutive 15 min intervals (0–15 to 105–120 min) and expressed as % of the time-matched control median. For each compound × concentration × time interval, outliers were removed using an IQR-based criterion (1.5× IQR) prior to calculating the median. Color intensity indicates locomotor activity (% control) as shown by the scale bar. The heatmap is intended as a descriptive visualization of temporal locomotor patterns across compounds and concentrations. Statistical comparisons for representative time windows were performed separately as described in Section 4.6 and are reported in Supplementary Table S3.

As shown in Figure 4, acute exposure of 5 dpf zebrafish eleutheroembryos to pyrrolidinophenone derivatives induced compound- and concentration-dependent decreases in BLA relative to controls. Locomotor activity was evaluated for all compounds at three concentrations (50 nM, 500 nM, and 5 µM) alongside vehicle controls and summarized at three representative time windows (0–15, 45–60, and 105–120 min), enabling comparison of early, intermediate, and late effects.

For α-PVP, exposure produced a marked reduction in BLA that was already evident during the first observation window and became more pronounced over time. Kruskal–Wallis analyses confirmed significant treatment effects at all three time windows: 0–15 min [H(3) = 61.90, *p* < 0.001], 45–60 min [H(3) = 24.81, *p* < 0.001], and 105–120 min [H(3) = 12.38, *p* = 0.006]. Post hoc Dunn comparisons indicated that, in the last observation window (105–120 min), only the highest concentration tested (5 μM) differed significantly from the control (*p* < 0.05), whereas lower concentrations had no detectable effect.

A similar but more potent pattern was observed for mono-chlorinated α-PVP derivatives (3-Cl-α-PVP and 4-Cl-α-PVP), which showed robust locomotor inhibition in all three observation windows. For 3-Cl-α-PVP, significant treatment effects were detected at 0–15 min [H(3) = 74.67, *p* < 0.001], 45–60 min [H(3) = 35.15, *p* < 0.001], and 105–120 min [H(3) = 19.77, *p* < 0.001], with post hoc analyses revealing significant reductions in BLA at 500 nM and 5 μM during the late phase (*p* < 0.05). Likewise, 4-Cl-α-PVP induced significant concentration-dependent decreases in locomotor activity at all time windows (0–15 min [H(3) = 93.79, *p* < 0.001], 45–60 min [H(3) = 45.17, *p* < 0.001], and 105–120 min [H(3) = 29.66, *p* < 0.001]), with sustained inhibition at intermediate and high concentrations.

MDPV and MDPiHP also produced significant locomotor inhibition, although their temporal profiles differed from those of the most potent PVP analogues. For MDPV, Kruskal–Wallis tests revealed significant treatment effects at 0–15 min [H(3) = 119.47, *p* < 0.001], 45–60 min [H(3) = 49.98, *p* < 0.001], and 105–120 min [H(3) = 53.86, *p* < 0.001], with post hoc analyses indicating that significant differences from controls were mainly driven by the 500 nM and 5 μM groups at later time points. MDPiHP displayed a comparable pattern, with significant effects at all three windows (all *p* < 0.001) and a clear dose-dependent reduction in BLA during the intermediate and late phases.

In contrast, 3,4-Cl-α-PVP exhibited a more moderate locomotor phenotype, with significant but less pronounced decreases in activity. Significant treatment effects were detected at 0–15 min [H(3) = 61.28, *p* < 0.001], 45–60 min [H(3) = 39.06, *p* < 0.001], and 105–120 min [H(3) = 22.47, *p* < 0.001], although post hoc comparisons indicated that reductions in BLA were primarily restricted to 500 nM and 5 μM at later time points.

Finally, α-PiHP and α-D2PV displayed comparatively lower locomotor potency, with significant treatment effects detected mainly at the highest concentration tested. For these compounds, Kruskal–Wallis analyses revealed significant effects at one or more observation windows (α-PiHP: 0–15 min [H(3) = 52.41, *p* < 0.001], 45–60 min [H(3) = 15.88, *p* = 0.001], 105–120 min [H(3) = 11.27, *p* = 0.010]; D2PV: 0–15 min [H(3) = 28.44, *p* < 0.001], 45–60 min [H(3) = 22.61, *p* < 0.001], 105–120 min [H(3) = 14.02, *p* = 0.003]), but post hoc analyses indicated that statistically significant reductions in BLA were largely confined to the 5 µM group. In contrast, 4-F-3-Me-α-PVP produced only modest locomotor suppression and did not achieve a ≥50% reduction in BLA within the tested concentration range.

To facilitate across-compound comparison of motor potency, concentration–response relationships were evaluated in the 105–120 min interval (Supplementary Figure S4; Supplementary Tables S1–S3). Locomotor EC_50_-like values were derived from pooled, replicate-level normalized data following per-condition outlier removal (1.5 × IQR), and were estimated by log-linear interpolation between the two concentrations (among 50 nM, 500 nM and 5 µM) that bracketed 50% residual activity (50% of the time-matched control). Using this late-phase approach, locomotor potency differed markedly between compounds, with α-PVP and chlorinated α-PVP analogs showing the lowest EC50-like values, whereas other derivatives required higher concentrations to approach half-maximal inhibition. Only 4-F-3-Me-α-PVP did not reach 50% inhibition at 5 µM, and its locomotor EC50-like value was therefore treated as not estimable within the tested range (right-censored; >5 µM) (Table 3).

### 2.4. Relationship Between Atrioventricular Block and Locomotor Inhibition

To integrate cardiac and neurobehavioral endpoints across the pyrrolidinophenone series, EC_50_ values for AV block were compared with EC_50_-like values for locomotor inhibition estimated in the 105–120 min interval. AV block was selected as a primary adverse cardiac endpoint, given its clear pathological relevance, whereas changes in atrial chronotropy may reflect physiological modulation within a normal functional range.

**Figure 5:**
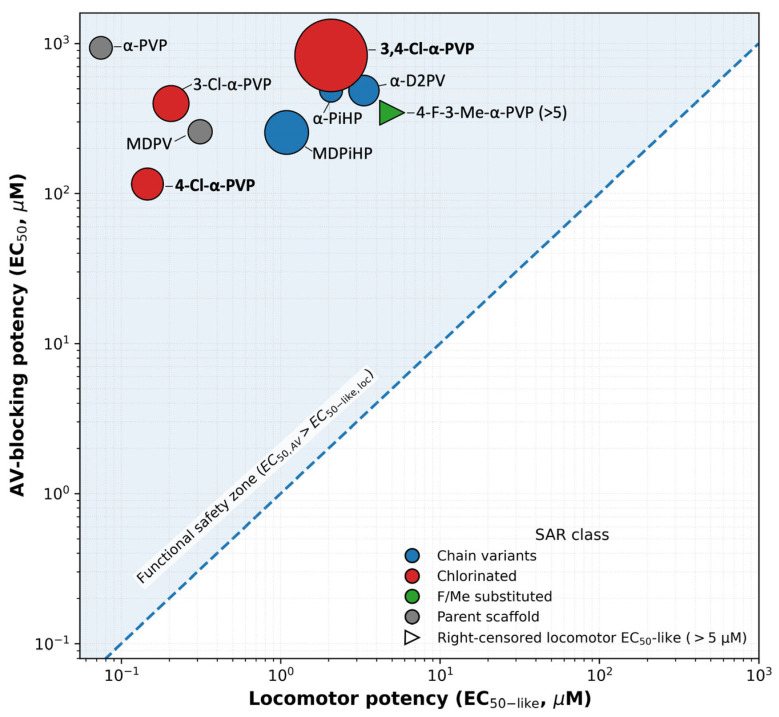
Cross-system bubble plot integrating neurobehavioral and cardiac potencies. Bubble plot showing the relationship between locomotor potency (EC_50_-like, µM; *x*-axis) and AV-blocking potency (EC_50_, µM; *y*-axis) for pyrrolidinophenone derivatives (log–log scale). Locomotor EC50-like values were derived from the 105–120 min interval by log-linear interpolation between concentrations bracketing 50% residual activity (50% of the time-matched control) after IQR-based outlier removal (1.5 × IQR). Bubble size encodes atrial chronotropic potency (chronotropy EC50, µM) expressed as pEC_50_ (−log(10) EC_50_); thus, larger bubbles indicate higher chronotropic potency (lower EC_50_). Bubble color indicates SAR class (chain variants, chlorinated, F/Me-substituted, and parent scaffolds). The dashed diagonal represents the identity line (y = x). The shaded region above the diagonal indicates the functional safety zone (EC_50_ AV block) > (locomotor EC_50_-like), where locomotor effects occur at lower concentrations than severe cardiac conduction impairment. For compounds not reaching 50% locomotor inhibition at 5 µM, locomotor EC_50_-like values are right-censored (>5 µM) and plotted at 5 µM (triangular marker).

As shown in Figure 5, comparison of EC_50_ values revealed pronounced compound-dependent differences in the relationship between AV block and locomotor effects. Most compounds exhibited AV block at concentrations substantially higher than those required to induce locomotor inhibition, indicating a wide separation between neurobehavioral and cardiac adverse effects. This pattern was particularly evident for α-PVP and chlorinated α-PVP derivatives (3-Cl-α-PVP and 4-Cl-α-PVP), which combined high motor potency with AV block EC_50_ values in the micromolar range.

MDPV and MDPiHP displayed intermediate profiles, with EC_50_-like values for locomotor inhibition in the submicromolar range and AV block occurring at concentrations approximately 2–3 orders of magnitude higher than those required to induce locomotor inhibition. In contrast, 3,4-Cl-PVP showed a reduced separation between endpoints, driven by its comparatively high AV block potency relative to its locomotor effects.

Finally, α-PiHP and α-D2PV showed comparatively low locomotor potency (higher EC_50_-like values), whereas 4-F-3-Me-α-PVP did not reach a 50% reduction in locomotor activity within the tested concentration range, resulting in a right-censored locomotor EC_50_-like (>5 µM) (Table 3; Figure 5). For this derivative, the degree of separation between neurobehavioral inhibition and AV block can therefore only be expressed as a bound rather than a precise ratio.

Overall, this integrative analysis highlights substantial heterogeneity within the pyrrolidinophenone family with regards to the relative sensitivity of neurobehavioral and cardiac adverse effects, providing a quantitative framework for subsequent interpretation in the Discussion.

## 3. Discussion

Zebrafish embryos are a well-established in vivo platform for detecting acute functional cardiotoxicity, including drug-induced bradyarrhythmia and conduction phenotypes that are relevant to repolarization and nodal dysfunction [20,21,22,23]. In particular, synthetic cathinones, such as MDPV, have been reported to induce chamber-specific rate effects, arrhythmia, and AV block in zebrafish, supporting the translational value of the model for NPS [19,24]. The present study provides a systematic functional analysis of the cardiac effects of a series of pyrrolidinophenone derivatives in zebrafish embryos, integrating quantitative analysis of atrial chronotropy and AV conduction. By applying a unified concentration–response modeling framework to both endpoints, our results highlight that minor structural modifications, such as halogenation and side-chain extension, can drastically alter their impact on cardiac rhythm and conduction in the zebrafish model. Moreover, our findings underscore a functional dissociation between atrial pacemaker activity and AV conduction, particularly evident in chlorinated analogues.

### 3.1. Structural Determinants of Atrial Chronotropic Effects

Bradycardia is often the earliest cardiovascular manifestation observed in zebrafish embryos exposed to psychoactive substances [19,24]. Since zebrafish express a hERG orthologue and demonstrate a sensitivity comparable to drugs that induce QT-interval prolongation in humans [22,23], atrial bradycardia serves as a robust functional surrogate for cardiac liability in this model [25]. While the chemical space explored in this study is limited and does not allow for a comprehensive structure–activity relationship (SAR) characterization, our results reveal specific structural alerts that differentiate cardiac liability from established neuropharmacological profiles.

Aromatic chlorination followed a pattern fundamentally distinct from that reported for monoamine transporters. In our series, the di-chlorinated analogue 3,4-Cl-α-PVP emerged as the most potent chronotropic agent (EC_50_ = 52.6 µM), exhibiting a six-fold increase in potency relative to unsubstituted α-PVP (EC_50_ = 323.9 µM). This finding is particularly striking when contrasted with neuropharmacological benchmarks: according to Nadal-Gratacós et al. (2022) [11], aromatic chlorination typically reduces DAT inhibition potency in vitro, with 3,4-Cl-α-PVP showing only intermediate activity. This discrepancy suggests that the atrial chronotropic effect in zebrafish may involve mechanisms not directly explained by dopamine transporter inhibition alone. Although the current lack of comparative data on norepinephrine transporter (NET) inhibition for these chlorinated derivatives precludes a definitive mechanistic assignment, our results support the view that the molecular determinants governing cardiac effects in the sinoatrial region may differ from those underlying dopaminergic activity.

The divergence between cardiac and neuropharmacological sensitivity is further highlighted by side-chain architecture. While the comparison between MDPV (EC_50_ = 303.4 µM) and MDPiHP (EC_50_ = 128.2 µM) appears to follow the “bulkiness” trend seen in mammalian DAT—where increased steric volume at the α-carbon side-chain correlates with higher biological potency [15], the case of α-PiHP (EC_50_ = 328.5 µM) breaks this rule in our model. Despite being a highly potent stimulant that induces robust locomotor and addictive effects in mammalian behavioral assays [11,26], α-PiHP was the least potent compound among the tested pyrrolidinophenones in the zebrafish heart. This mismatch reinforces the idea that cardiac liability cannot be inferred directly from neurochemical potency alone, as modifications that enhance a “high” in the central nervous system may, in some cases, result in a relatively lower risk of acute atrial dysfunction, and vice versa.

### 3.2. Structural Determinants of AV Conduction Block

In contrast to atrial chronotropy, AV conduction block displayed a distinct SAR. Several compounds with strong atrial chronotropic effects produced little or no AV block, whereas others preferentially impaired AV conduction at concentrations associated with modest atrial slowing. This divergence suggests that the determinants of AV node conduction may be at least partly distinct from those governing pacemaking mechanisms. In zebrafish, AV conduction depends on a physiological delay regulated by L-type calcium channels (I*_Ca,L_*) and voltage-gated sodium channels (I*_Na_*), whose roles in zebrafish show notable functional homology to those in humans [27,28].

Among the compounds tested, 4-Cl-α-PVP showed the lowest point estimate for AV-blocking potency. Notably, this pattern was observed despite only moderate effects on atrial rate, suggesting preferential AV-conduction liability within the limits of the present dataset. One possible interpretation is that the para-chloro substituent may influence molecular targets involved in AV impulse propagation, potentially including sodium- or calcium-channel–dependent mechanisms, although this cannot be resolved within the scope of the present study [29]. Notably, α-PVP exhibited very weak AV-blocking potency (EC_50_ = 937.0 µM), while also showing only modest atrial chronotropic effects (EC_50_ = 323.9 µM), underscoring the weak coupling between these two cardiac endpoints.

In contrast, 3-Cl-α-PVP and especially 3,4-Cl-α-PVP displayed relatively weak AV-blocking activity despite their strong chronotropic effects. This pattern suggests that aromatic chlorination does not uniformly affect all cardiac endpoints; rather, the position of the substitution may influence whether the molecule preferentially affects pacemaker- or conduction-related processes. While positional sensitivity is a known factor in DAT affinity [11], our findings are consistent with the possibility that cardiac conduction is influenced by pharmacological determinants distinct from those driving dopaminergic potency. The lack of a clear correspondence between DAT inhibition and these cardiac endpoints is consistent with the involvement of alternative pathways, such as the modulation of specific cardiac ion channels or adrenergic receptors, where the SAR does not parallel that of the dopaminergic system.

Finally, the comparison between MDPV and MDPiHP reinforces the independence of these two cardiac effects, as both showed similar AV-blocking potencies despite their chronotropic differences. While side-chain bulk is a driver of neurochemical potency, it appears to play a less decisive role in AV conduction. The fact that branched analogues like α-PiHP possess high behavioral potency [26], but low AV-blocking capacity, supports the view that NPS cardiotoxicity is a multidimensional phenomenon that requires integrated in vivo models for accurate risk assessment. These apparent structure-related differences should be interpreted with appropriate caution, particularly where AV-block estimates were associated with broader confidence intervals.

### 3.3. Functional Dissociation Between Atrial Chronotropy and AV Block

When atrial chronotropy and AV conduction are considered together, a clear functional dissociation emerges. Across the entire series of compounds, neither endpoint can be reliably predicted from the other, and several analogues display preferential effects on one cardiac function over the other. For example, 3,4-Cl-α-PVP showed strong atrial chronotropic potency (EC_50_ = 52.6 µM) while remaining a comparatively weak AV-blocking agent (EC_50_ = 833.5 µM), consistent with preferential impairment of atrial pacemaker function. In contrast, 4-Cl-α-PVP preferentially disrupted AV conduction (EC_50_ = 115.8 µM) despite its only moderate chronotropic potency, indicating functional selectivity toward atrioventricular conduction liability. α-PVP exhibited modest chronotropic potency (EC_50_ = 323.9 µM) together with very weak AV-blocking potency (EC_50_ = 937.0 µM), further underscoring that atrial pacemaker inhibition and AV conduction block are not tightly coupled within this series. Other compounds, such as 4-F-3-Me-α-PVP, occupied an intermediate position, with atrial and AV effects occurring within a similar concentration range.

These findings suggest that AV block is not simply a downstream consequence of atrial slowing or generalized cardiodepression. Instead, atrial pacemaker inhibition and AV nodal conduction block represent distinct functional endpoints, each shaped by specific SAR constraints. Independent modulation of rhythm and conduction has been noted in zebrafish embryos exposed to QT-prolonging drugs, where early bradycardia and AV block can appear as partially decoupled phenotypic markers [22,23].

The observed pattern raises the possibility that the para-chloro substituent in 4-Cl-PVP may preferentially influence targets involved in impulse propagation through the atrioventricular canal (AVC). In zebrafish, the AVC serves as a functional bottleneck where conduction is significantly slower than in the chambers, relying heavily on a specific balance of L-type Ca^2+^ currents and Na^+^ channel kinetics [30]. Perturbation of these pathways by diverse toxicants often manifests as changes in the conduction ratio (e.g., 2:1 block) at exposures that do not necessarily produce marked reductions in atrial firing rate.

Moreover, the high atrial chronotropic potency of 3,4-Cl-α-PVP is consistent with the recognized sensitivity of zebrafish cardiac rhythm to perturbations affecting repolarization. As described in zebrafish electrophysiological and pharmacological studies [31,32], this model displays a human-like repolarization profile and can exhibit early negative chronotropy in response to compounds with QT-prolonging potential, supporting the interpretation that atrial chronotropy can serve as an early functional indicator of liability in vivo.

By capturing these distinct physiological “fingerprints,” our study reinforces the utility of multiparametric dynamic pixel analysis as a New Approach Methodology (NAM). This approach moves beyond simple heart rate counting, common in earlier zebrafish screens [33], to provide a multiparametric characterization of NPS cardiotoxicity that is informative for translational risk assessment [24]. Nevertheless, differences between zebrafish and human cardiovascular physiology, as well as differences in exposure conditions and pharmacokinetics, limit direct extrapolation of these findings to human cardiovascular risk. Moreover, a methodological limitation of this cardiac assay is the brief use of tricaine prior to imaging, which may influence absolute cardiac rate measurements in zebrafish embryos and early larvae. However, because all groups were recorded under the same standardized conditions, this factor is unlikely to account for the differential treatment-related effects observed across compounds.

### 3.4. Implications for Cardiotoxicity and Structure-Based Risk Assessment

The dissociation observed between atrial chronotropy and AV conduction has important implications for the interpretation of cardiotoxic risk. Compounds that predominantly depress atrial pacemaker activity may predispose to bradycardic phenotypes consistent with sinoatrial dysfunction, whereas AV-selective compounds, exemplified here by 4-Cl-α-PVP, can induce high-grade conduction disturbances even when atrial activity is only modestly affected. These divergent phenotypes are consistent with the functional compartmentalization of rhythm generation and impulse propagation within the cardiac syncytium, processes governed by partially distinct sets of ion channels and intercellular coupling mechanisms [25].

From a structure–activity perspective, our results emphasize the necessity of multiparametric functional readouts. Many earlier zebrafish-based screens relied primarily on single endpoints such as heart rate or lethality, approaches that would not capture the qualitative differences observed across this pyrrolidinophenone series [33]. For example, prioritizing AV block alone would underestimate the pronounced atrial chronotropic liability of 3,4-Cl-α-PVP, whereas reliance on atrial rate alone would fail to identify compounds with preferential conduction liability. Together, these findings support the view that NPS cardiotoxicity is not a uniform “class effect” but rather a spectrum of structure-dependent dysfunctions affecting distinct electrophysiological processes.

Furthermore, the integration of these findings into a NAM framework aligns with current regulatory shifts towards more mechanistically informative assays [24]. As the zebrafish embryo possesses a repolarization profile and an electrophysiological repertoire that closely mimics human physiology—including the presence of the KCNH2 (zERG) ortholog—these SAR constraints are likely translatable to human clinical risks [24,31].

**Figure 6:**
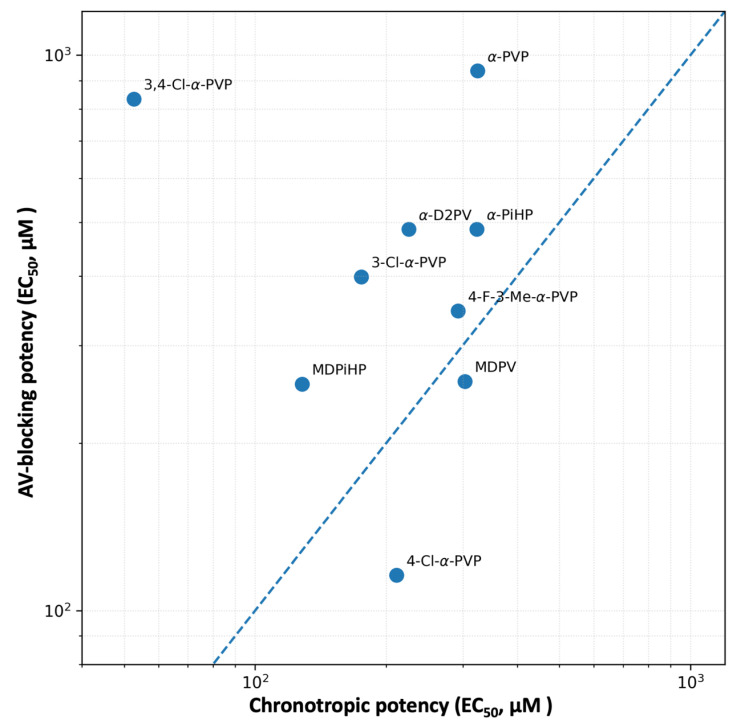
Dissociation between atrial chronotropy and AV conduction block. Relationship between atrial chronotropic potency (EC_50_ for atrial rate inhibition) and AV-blocking potency (EC_50_ for AV block) for the tested pyrrolidinophenone derivatives in zebrafish embryos. Each dot represents one compound. The dashed diagonal line indicates equal potency for both endpoints. Points above the diagonal indicate greater potency for atrial rate inhibition than for AV block (EC50_AV block > EC50_chronotropy), whereas points below the diagonal indicate relatively greater AV-blocking potency (EC50_AV block < EC50_chronotropy). Compounds deviating from the diagonal illustrate functional selectivity toward atrial pacemaker inhibition or AV conduction impairment. EC_50_ values were derived from four-parameter logistic models fitted to replicate-level data at concentrations ≥ 5 µM. This figure is intended as a comparative visualization of endpoint separation across compounds and does not imply a specific molecular mechanism.

The functional dissociation between atrial chronotropy and AV conduction is summarized in Figure 6, which illustrates the lack of a linear relationship between chronotropic and AV-blocking potency across the tested compounds. Deviations from the line of equipotency highlight compounds with selective liability toward either atrial pacemaker inhibition or AV conduction impairment, underscoring that subtle structural modifications can markedly shift cardiac risk profiles. The combined assessment of atrial chronotropy and AV conduction therefore offers a more comprehensive framework for structure-based cardiotoxicity assessment of synthetic stimulants [32]. Although extrapolation to human exposure scenarios requires caution, the concentration–response relationships observed in zebrafish provide mechanistic insight that can inform hypothesis-driven follow-up studies and improve prioritization of emerging pyrrolidinophenone analogues.

### 3.5. Integration with Locomotor Endpoints: Time-Course, Potency, and Functional Safety Index

Time-resolved locomotor profiling revealed that pyrrolidinophenone derivatives differ not only in the magnitude of motor activity suppression but also in their onset and persistence. Potent analogues, such as α-PVP and its mono-chlorinated derivatives, produced rapid hypoactivity that was apparent within the initial 15 min and remained consistent throughout the late phases of the assay. In contrast, other derivatives, particularly those with bulkier substitutions, exhibited delayed or modest effects that only reached statistical significance in later observation windows.

This temporal heterogeneity is consistent with the dynamic behavioral responses previously reported for MDPV in zebrafish eleutheroembryos, where initial acclimation periods can mask the full extent of drug-induced impairment [19]. Furthermore, the observation of hypoactivity at high-micromolar concentrations in zebrafish, rather than the hyperlocomotion typically seen in rodent models at lower doses, likely reflects a transition from psychostimulant activity to systemic neurotoxicity or a “behavioral shutdown” induced by massive monoaminergic elevations, a phenomenon characteristic of potent uptake inhibitors [8,19]. Reduced locomotor activity should be interpreted as a functional neurobehavioral readout within the assay, particularly in the context of pyrrolidinophenones with known psychostimulant activity. However, this phenotype is not mechanistically specific and may also reflect sedation-like effects, motor impairment, or broader systemic toxicity.

To facilitate across-compound comparison, motor potency was quantified during the 105–120 min interval, a late-phase window less influenced by transient acclimation effects. Based on EC_50_-like estimates, the series could be stratified into: (I) high-potency locomotor inhibitors (submicromolar EC50-like), including α-PVP, 3-Cl-α-PVP, 4-Cl-α-PVP, and MDPV; (II) intermediate-potency derivatives (low micromolar EC50-like), including MDPiHP, 3,4-Cl-α-PVP, α-PiHP, and α-D2PV; and (III) a right-censored low-potency compound, 4-F-3-Me-α-PVP, which did not reach a 50% reduction within the tested concentration range (EC50-like >5 µM).

These findings demonstrate that subtle modifications within the pyrrolidinophenone scaffold—such as the shift from a methylenedioxy group to a single halogen—can markedly increase neurobehavioral potency in vivo. This aligns with reported SAR studies where the length of the alkyl chain (e.g., the five-carbon valerophenone chain) and specific ring substitutions optimize inhibition at DAT [11,15,34].

Finally, integrating locomotor potency with atrioventricular (AV) block concentration–response relationships provided a comparative measure of the functional separation between psychostimulant-like locomotor effects and a severe adverse cardiac endpoint within the same comparative zebrafish study framework. For most tested derivatives, AV block occurred at substantially higher concentrations than those required to reduce locomotion, indicating broad separation between both functional domains in the zebrafish model. Across the series, these separations typically spanned approximately 2–4 orders of magnitude, depending on the compound. However, this separation was notably narrower for 4-Cl-α-PVP, which emerged as a compound of comparatively greater concern within the present dataset because of its pronounced AV-blocking liability at concentrations closer to those producing locomotor effects.

By contrast, the wider separation observed for α-PiHP within this assay suggests a lower degree of overlap between psychostimulant-like locomotor effects and severe AV-conduction toxicity. However, this should not be interpreted as absence of cardiovascular risk through other mechanisms, since sympathomimetic manifestations such as tachycardia, vasoconstriction, and hypertension have been described in clinical and forensic reports for related pyrrolidinophenones [16,17,18,35]. Conceptually, the Functional Safety Index reflects the extent to which psychostimulant-like and cardiotoxic effect domains remain separated within the assay. A broader separation suggests a lower probability that exposures associated with prominent psychostimulant-like effects will also approach a range producing severe cardiac conduction toxicity, whereas a narrower separation indicates greater potential overlap between both domains. At present, this interpretation remains assay-specific and comparative, since mammalian and human exposure–effect data allowing direct translational calibration are currently very limited or unavailable for these compounds. Accordingly, the FSI should not be understood as a direct predictor of human safety, but rather as a heuristic comparative descriptor that may help prioritize compounds for further cardiotoxicological evaluation. This interpretation should also be considered in light of the lower concentration resolution of the locomotor assay relative to the cardiac concentration–response analysis.

### 3.6. Pharmacological Alignment: From Molecular DAT Inhibition to in Vivo Locomotor Impairment

A critical finding of this study is the high degree of consistency between the locomotor effects observed in zebrafish and the established SAR for DAT inhibition. The pyrrolidinophenone scaffold is characterized by its action as a potent, non-substrate blocker of monoamine transporters [15,19]. Our results reinforce the notion that the neurobehavioral potency of these derivatives is strictly dictated by specific structural motifs on the phenyl ring and the alkyl chain.

The observation that α-PVP and several close analogues (including 3-Cl-α-PVP, 4-Cl-α-PVP and MDPV) were among the most potent inhibitors of locomotor activity in our model aligns with in vitro data showing IC_50_ values for DAT in the low nanomolar range (12–18 nM) [11,15]. Interestingly, our data reflect a key SAR principle: the introduction of halogens generally preserves or enhances lipophilicity, facilitating CNS penetration and transporter binding. As reported by Nadal-Gratacós et al. (2022) [11], while meta-substitution (e.g., 3-Cl-α-PVP) tends to increase the DAT/SERT selectivity ratio, para-substitution (e.g., 4-Cl-α-PVP) maintains extreme potency as a blocker [11]. The rapid onset of hypoactivity observed in our time-course analysis for these chlorinated analogs suggests an immediate and massive elevation of synaptic DA, leading to the “behavioral titration” or shutdown typically seen in zebrafish at micromolar concentrations [19].

Our results also validate the “valerophenone rule” established by Kolanos et al. (2015), which identifies the five-carbon α-alkyl chain as the optimal length for DAT inhibition within the pyrrolidinophenone series [15]. Compounds with this backbone, such as α-PVP and its chlorinated derivatives, exhibited higher potency in suppressing locomotion than derivatives with bulkier side-chain variants. In particular, the lower locomotor potency observed for α-PiHP and α-D2PV in our zebrafish model is consistent with reports that increasing chain length or introducing steric bulk can reduce DAT affinity by impairing optimal accommodation within the transporter binding pocket [10,15,17].

While hyperlocomotion is the hallmark of low-dose psychostimulant action in rodents, the sustained hypoactivity recorded in our eleutheroembryos at 500 and 5 μM serves as a functional readout of “extreme” DAT blockade. This dose-dependent inversion of the locomotor response is a known feature of the zebrafish model for potent uptake inhibitors like MDPV [19]. By showing that the rank order of locomotor suppression in zebrafish closely follows the rank order of DAT IC_50_ values reported in the literature, we provide strong evidence that this model can accurately prioritize the neuropharmacological hazard of newly emerging NPS based on their primary molecular mechanism.

### 3.7. Conclusions

This study provides a comprehensive, integrated characterization of the neuro-cardiotoxic profiles of second-generation pyrrolidinophenone derivatives. By moving beyond mortality-based endpoints and applying a multiparametric NAM approach, we characterized the functional relationship between neurobehavioral potency and cardiac liability.

Our findings identify a robust functional dissociation between atrial chronotropy and AV conduction, whereby minor structural modifications, particularly aromatic chlorination, shape the dominant cardiac phenotype. While 3,4-dichlorination (3,4-Cl-α-PVP) maximized negative chronotropic potency, para-monochlorination (4-Cl-α-PVP) biased the profile toward AV conduction failure. Crucially, integrating these results with locomotor activity data enabled a Functional Safety Index that highlights the compound-specific degree of functional separation between neurobehavioral inhibition and severe cardiac conduction impairment within this assay. While most derivatives exhibited wide separations between endpoints, 4-Cl-α-PVP emerged as a higher-priority compound within the present dataset because of its comparatively reduced endpoint separation driven by AV-blocking potency.

More broadly, our results reinforce the utility of the zebrafish embryo as a multiparametric platform for comparative hazard prioritization. The ability to resolve distinct physiological transitions, such as the emergence of 2:1 AV block in the presence of relatively preserved atrial activity, demonstrates that rhythm and conduction liabilities can be uncoupled even within closely related scaffolds. Future studies integrating these in vivo phenotypes with targeted ion-channel assays or calcium imaging will be essential to unravel molecular determinants (e.g., hERG or Nav channel inhibition) underlying this selectivity.

Finally, these findings may have relevance for clinical and forensic toxicology. Our data indicate that neurobehavioral potency does not necessarily scale linearly with cardiac liability, and that chronotropic changes alone may not predict severe conduction disturbances. Accordingly, the absence of marked bradycardia should not be assumed to exclude AV conduction risk. Incorporating endpoint-resolved functional signatures into structure-based predictive frameworks may improve early comparative risk assessment in the evolving NPS landscape, although extrapolation to human exposure scenarios should be made with caution.

## 4. Materials and Methods

### 4.1. Zebrafish Maintenance and Embryo Collection

Adult wild-type short-fin zebrafish (*Danio rerio*) used as breeders were housed at the CID-CSIC (Barcelona, Spain) in a recirculating aquatic system (Aquaneering Inc., San Diego, CA, USA) under standard conditions (28 ± 1 °C; 12 h:12 h light/dark photoperiod). Breeding groups (three females and two males) were placed in spawning tanks the day before each experiment. Spawning was induced at lights-on and fertilized eggs were collected within 30 min. Only morphologically normal embryos at the 50% epiboly stage were selected under a stereomicroscope and maintained in embryo water (Milli-Q water supplemented with Instant Ocean salts and CaSO_4_·2H_2_O; pH 6.5–7.0; conductivity 750–900 µS/cm) at 28 ± 1 °C until 3 days post-fertilization (3 dpf) for cardiac assays, and to 5 dpf for locomotor assays, as specified in the corresponding sections below. All procedures were approved by the Institutional Animal Care and Use Committees at CID-CSIC and conducted under local governmental authorization (agreement number 11336).

### 4.2. Compounds and Exposure Solutions

The pyrrolidinophenone derivatives (α-PVP, α-PiHP, α-D2PV, 3-Cl-α-PVP, 4-Cl-α-PVP, 3,4-α-Cl-PVP, 4-F-3-Me-α-PVP, MDPV, and MDPiHP) were synthesized in racemic form as hydrochloride salts and characterized as described in the Supplemental Material. Chemical structures are shown in Figure 6. Stock solutions (10 mM) were freshly prepared in Milli-Q water on each experimental day, and working solutions were obtained by dilution in embryo water. When needed, pH was adjusted with 1 M NaHCO_3_ to keep solutions in an alkaline range compatible with pyrrolidine-containing cathinones.

### 4.3. Cardiotoxicity Assessment

At 3 dpf, embryos were exposed for 2 h to control (0 µM) or to 5, 50, 100, 200, or 400 µM of each compound in 48-well plates (one embryo per well; 1 mL per well). Each embryo was treated as an independent biological replicate for cardiac endpoint analysis. After exposure, embryos were immediately processed for cardiac recordings. Embryos lacking a heartbeat were considered dead and excluded from functional analyses; in the raw datasheets, missing values due to mortality were annotated as “x”. At the highest concentration tested (400 µM), acute mortality was observed during the 2-h exposure window for 3,4-Cl-PVP, 4-F-3-Me-PVP, 4-Cl-PVP and 3-Cl-PVP; therefore, cardiac endpoint estimates at 400 µM reflect only surviving embryos (Dataset S1).

To minimize movement during imaging, 3 dpf embryos were briefly anesthetized with tricaine methane–sulfonate (MS-222; 0.08 mg/mL, corresponding to 0.008%) for approximately 10 s and embedded in 3% methylcellulose on a depression slide. This concentration and short exposure duration were selected to enable transient immobilization during image acquisition and are within the range reported in the zebrafish literature for brief cardiac imaging procedures [36,37,38,39,40,41]. However, because tricaine may affect cardiac physiology in a concentration-, developmental stage-, and exposure-dependent manner, its potential influence on absolute cardiac values should be considered when interpreting the data.

Cardiac activity was recorded laterally at 28 ± 1 °C using a stereomicroscope coupled to a high-speed camera (100 frames/s; 20 s) in our temperature-controlled behavioral room, where ambient temperature was maintained at 28 ± 1 °C during image acquisition. Because all experimental groups were subjected to the same brief immobilization procedure, any tricaine-related effects would be expected to act as a common procedural background rather than a treatment-specific bias.

Atrial and ventricular beat frequencies (beats/min) were quantified using DanioScope (Noldus IT, Wageningen, The Netherlands), which derives rhythmicity from pixel-intensity fluctuations over time. The atrioventricular (AV) ratio was calculated as the atrial rate divided by the ventricular rate (R = A/V).

AV conduction failure was expressed as percentage AV block using the following:

AV block (%) = 100 × (1 − 1/R)(1)

which maps normal conduction (R = 1) to 0% and complete block (ventricular activity approaching zero) to 100%. Negative chronotropy was expressed as percent inhibition of atrial rate relative to the matched control within each embryo:

Chronotropy (%) = 100 × (1 − A_treated_/A_control_). (2)

Chronotropy values were clipped to the 0–100% range to match bounded-response modeling. Concentration–response relationships for chronotropy and AV block were modeled using replicate-level data, as described in Section 4.6.

### 4.4. Behavioral Assessment

Basal locomotor activity (BLA) was assessed in 5 dpf zebrafish eleutheroembryos exposed for 2 h to pyrrolidinophenone derivatives at nominal concentrations of 50 nM, 500 nM, and 5 μM. Untreated embryo water served as the control. Recordings were performed using the DanioVision Observation Chamber (Noldus IT, Wageningen, The Netherlands) under near-infrared illumination, with temperature maintained at 28 °C. Video acquisition was carried out using EthoVision XT software (v13 for recording, v16 for analysis).

Locomotor behavior was quantified continuously throughout the 120 min exposure period using consecutive 15 min bins (0–15 to 105–120 min). For each eleutheroembryo, BLA was defined as the total distance traveled (cm) within each observation window. For primary statistical comparisons and for concise reporting of early, intermediate, and late effects, three representative windows were predefined (0–15, 45–60, and 105–120 min). For visualization of full behavioral kinetics throughout the exposure period, all eight consecutive 15 min intervals were summarized in heatmap form.

Experiments were conducted in three independent batches, each including 12 eleutheroembryos per condition. Each eleutheroembryo was treated as an independent biological replicate. To account for inter-batch variability, locomotor data from each experiment were normalized to the median BLA of the corresponding control group, which was set to 100%, prior to pooling data across experiments. All subsequent analyses were therefore performed on normalized values, resulting in 36 eleutheroembryos per condition.

To facilitate across-compound comparison of motor potency, late-phase locomotor activity (105–120 min) was summarized using pooled, replicate-level normalized data. This interval was selected to capture sustained effects under continuous exposure. Outliers were removed per condition using an IQR-based criterion (1.5 × IQR), applied as an objective and standardized data-cleaning approach. However, recordings associated with outlier values were not systematically re-inspected individually, and therefore the contribution of biological variability versus potential tracking artifacts could not be determined. Locomotor EC50-like values were estimated from the late-phase (105–120 min) normalized responses by log-linear interpolation between the two tested concentrations (among 50 nM, 500 nM, and 5 µM) that bracketed 50% residual activity. Because the behavioral assay was designed for time-resolved multi-compound screening using three concentrations, full logistic concentration–response modeling was not considered sufficiently robust for locomotor data; therefore, potency was expressed as EC50-like values derived by interpolation. When 50% inhibition was not reached within the tested range (≤5 µM), EC_50_-like values were reported as not estimable (not reached within 5 µM). Outlier removal rates are summarized in Supplementary Figure S1, and median-based late-phase summary statistics and EC50-like estimates are provided in Supplementary Tables S1 and S2.

### 4.5. Safety Index Calculation

To quantify the functional separation between neurobehavioral effects and adverse cardiac conduction disturbances among compounds, a Functional Safety Index (FSI) was calculated as the ratio between the EC50 for AV block and EC_50_-like for locomotor inhibition:

(3) $$FSI=\frac{{\mathrm{EC}}_{50}\mathrm{AV}\;\mathrm{block}}{{\mathrm{EC}}_{50}-\mathrm{like}\;\mathrm{locomotor}}$$

where EC_50_ AV block corresponds to the half-maximal effective concentration for AV block estimated from the concentration–response analysis of cardiac endpoints, and locomotor EC_50_-like corresponds to the EC_50_-like estimate derived from the 105–120 min locomotor interval as described above. Conceptually, this ratio is intended to capture the degree of separation between a psychostimulant-like functional domain and a severe cardiotoxic endpoint measured in complementary zebrafish assays within the same comparative study framework. In this sense, the FSI follows the general logic of ratio-based pharmacological indices relating functional activity to toxicity or safety margin [42], but here it is used only as an assay-specific comparative descriptor within a uniform zebrafish testing framework. Accordingly, the FSI is not intended as a universal toxicological constant or as a direct predictor of human clinical safety. Because the locomotor component is based on EC50-like estimates rather than model-derived EC50 values, the FSI should be interpreted as a comparative within-study descriptor rather than as a precise pharmacodynamic ratio. When 50% locomotor inhibition was not reached within the tested range (≤5 µM), locomotor EC_50_-like was treated as right-censored (>5 µM) and FSI was reported as an upper bound: FSI < (EC_50_ AV block)/5. FSI values were used for comparative visualization and across-compound ranking.

### 4.6. Statistical Analysis

Data were analyzed with IBM SPSS v29 (Statistical Package 2010, Chicago, IL, USA). Normality of the data distribution was assessed using the Shapiro–Wilk test. Because the assumption of normality was not met, statistical significance was evaluated using the Kruskal–Wallis test, followed by Dunn–Bonferroni post hoc comparisons for multiple-group analyses. Statistical significance was set at *p* < 0.05.

## Figures and Tables

**Table 1:** Chronotropic effects: EC50 and Hill slope values. EC_50_ and Hill slope values were obtained from four-parameter logistic (4PL) concentration–response models constrained between 0 and 100% and fitted to individual replicate data at concentrations ≥ 5 µM. Chronotropic effect was expressed as percent decrease in atrial heart rate relative to the matched control within each embryo (100 × (1 − atrial/control)), clipped to 0–100%. Parameter uncertainty was assessed by nonparametric bootstrap resampling (*n* = 2000). Values are reported as estimates with 95% confidence intervals. n indicates the number of embryos contributing data at each concentration. Compounds are ordered according to increasing atrial chronotropic potency (ascending EC_50_ values).

Compound	EC_50_ (µM)	95% CI (EC_50_)	Hill Slope	95% CI (Hill)
3,4-Cl-α-PVP	52.6	46.3–60.4	0.98	0.89–1.08
MDPiHP	128.2	116.4–140.0	0.84	0.76–0.94
3-Cl-α-PVP	175.2	156.4–197.6	0.95	0.85–1.09
4-Cl-α-PVP	211.1	183.8–241.6	0.79	0.70–0.90
α-D2PV	225.4	208.2–244.7	1.51	1.36–1.68
4-F-3-Me-α-PVP	292.5	257.1–331.5	0.94	0.84–1.08
MDPV	303.4	263.9–347.1	0.88	0.77–1.01
α-PiHP	323.0	302.9–342.4	2.14	1.84–2.56
α-PVP	323.9	284.3–375.0	1.09	0.95–1.25

**Table 2:** EC50 and Hill slope values for AV block induction. EC_50_ and Hill slope values were obtained from four-parameter logistic (4PL) concentration–response models constrained between 0 and 100% and fitted to individual replicate data at concentrations ≥ 5 µM. Parameter uncertainty was assessed by nonparametric bootstrap resampling (*n* = 2000). Values are reported as estimates with 95% confidence intervals. Compounds are ordered according to increasing AV-blocking potency (ascending EC_50_ values).

Compound	EC_50_ (µM)	95% CI (EC_50_)	Hill Slope	95% CI (Hill)
4-Cl-α-PVP	115.8	94.3–137.7	1.31	1.00–1.76
MDPiHP	255.6	212.7–311.6	1.02	0.80–1.30
MDPV	258.5	222.6–307.5	1.36	1.08–1.76
4-F-3-Me-α-PVP	346.1	298.4–387.5	3.04	2.30–4.94
3-Cl-α-PVP	398.9	327.8–526.3	1.86	1.39–2.64
α-D2PV	485.6	446.6–566.5	10.00	9.92–10.00
α-PiHP	486.0	446.6–567.5	10.00	9.91–10.00
3,4-Cl-α-PVP	833.5	350.3–6300.2	0.52	0.31–0.88
α-PVP	937.0	463.0–10,727.9	2.29	0.87–10.00

**Table 3:** Summary of potency parameters and Functional Safety Index (SI). Locomotor EC_50_-like values were obtained in the 105–120 min interval by log-linear interpolation between concentrations bracketing 50% residual activity after IQR-based outlier removal (1.5 × IQR). When 50% inhibition was not reached within the tested range (≤5 µM), EC_50_-like was reported as >5 µM, and FSI as < EC_50_ AV block/5.

Compound	EC_50_ Atrial (µM)	EC_50_ AV Block (µM)	Locomotor EC_50_-Like (µM)	Functional Safety Index
MDPV	303.4	258.5	0.3125	827.11
MDPiHP	128.2	255.6	1.088	235.02
α-PVP	323.9	937.0	0.07449	12578.52
α-D2PV	225.4	485.6	3.33	145.81
α-PiHP	323.0	486.0	2.069	234.95
3-Cl-α-PVP	175.2	398.9	0.2049	1946.86
4-Cl-α-PVP	211.1	115.8	0.1459	793.49
3,4-Cl-α-PVP	52.6	833.5	2.068	402.98
4-F-3-Me-α-PVP	292.5	346.1	>5	<69.22

## Data Availability

The data supporting the findings of this study are available within the manuscript and its Supplementary Material File or will be made available by the corresponding author upon request.

## Supplementary Materials

The following supporting information can be downloaded at: https://www.mdpi.com/article/10.3390/ijms27073141/s1. Reference [43] was cited in the Supplementary File.

## References

[1] United Nations Office on Drugs and Crime What Are NPS?. https://www.unodc.org/LSS/Page/NPS.

[2] United Nations Office on Drugs and Crime Current NPS Threats. https://sway.cloud.microsoft/SBdzGtMZGnxpGRRA?ref=Link.

[3] Oliver C.F., Palamar J.J., Salomone A., Simmons S.J., Philogene-Khalid H.L., Stokes-McCloskey N., Rawls S.M. (2019). Synthetic cathinone adulteration of illegal drugs. Psychopharmacology.

[4] Chen S., Zhou W., Lai M. (2024). Synthetic cathinones: Epidemiology, toxicity, potential for abuse, and current public health perspective. Brain Sci..

[5] Zaami S., Giorgetti R., Pichini S., Pantano F., Marinelli E., Busardò F.P. (2018). Synthetic cathinones related fatalities: An update. Eur. Rev. Med. Pharmacol. Sci..

[6] European Union Drugs Agency (2024). New Psychoactive Substances—The Current Situation in Europe (European Drug Report 2024).

[7] European Union Drugs Agency (2025). New Psychoactive Substances—The Current Situation in Europe (European Drug Report 2025).

[8] Nadal-Gratacós N., Pazos M.D., Pubill D., Camarasa J., Escubedo E., Berzosa X., López-Arnau R. (2024). Structure–activity relationship of synthetic cathinones: An updated review. ACS Pharmacol. Transl. Sci..

[9] Kolaczynska K.E., Thomann J., Hoener M.C., Liechti M.E. (2021). The pharmacological profile of second generation pyrovalerone cathinones and related cathinone derivative. Int. J. Mol. Sci..

[10] Nadal-Gratacós N., Mata S., Puigseslloses P., De Macedo M., Lardeux V., Pain S., Wang F.-H., Källsten L., Pubill D., Berzosa X. (2025). Unveiling the potential abuse liability of α-D2PV: A novel α-carbon phenyl-substituted synthetic cathinone. Neuropharmacology.

[11] Nadal-Gratacós N., Lleixà E., Gibert-Serramià M., Estrada-Tejedor R., Berzosa X., Batllori X., Pubill D., Camarasa J., Escubedo E., López-Arnau R. (2022). Neuropsychopharmacology of emerging drugs of abuse: Meta- and para-halogen-ring-substituted α-PVP (“flakka”) derivatives. Int. J. Mol. Sci..

[12] Kriikku P., Ojanperä I. (2024). Findings of synthetic cathinones in post-mortem toxicology. Forensic Sci. Int..

[13] Pieprzyca E., Skowronek R., Czekaj P. (2022). Toxicological analysis of intoxications with synthetic cathinones. J. Anal. Toxicol..

[14] Riley A.L., Nelson K.H., To P., López-Arnau R., Xu P., Wang D., Wang Y., Shen H., Kuhn D.M., Angoa-Perez M. (2020). Abuse potential and toxicity of the synthetic cathinones (i.e., “Bath salts”). Neurosci. Biobehav. Rev..

[15] Kolanos R., Sakloth F., Jain A.D., Partilla J.S., Baumann M.H., Glennon R.A. (2015). Structural modification of the designer stimulant α-pyrrolidinovalerophenone (α-PVP) influences potency at dopamine transporters. ACS Chem. Neurosci..

[16] Zawilska J.B., Wojcieszak J. (2013). Designer cathinones—An emerging class of novel recreational drugs. Forensic Sci. Int..

[17] Brueckner I., Welter-luedeke J., Zangl A., Graw M., Paul L.D. (2024). α-Pyrrolidinohexanophenone (α-PHP) vs α-pyrrolidinoisohexanophenone (α-PiHP): A toxicological investigation about plasma concentrations and behavior in forensic routine cases. J. Anal. Toxicol..

[18] Dinis P., Franco J., Margalho C. (2024). α-Pyrrolidinohexanophenone (α-PHP) and α-pyrrolidinoisohexanophenone (α-PiHP): A Review. Life.

[19] Aljabasini O., Tagkalidou N., Bedrossiantz J., Prats E., Arnau R.L., Raldua D. (2025). Integrated assessment of the cardiotoxic and neurobehavioral effects of 3,4-methylenedioxypyrovalerone (MDPV) in zebrafish embryos. Int. J. Mol. Sci..

[20] Stainier D.Y.R., Fishman M.C. (1994). The zebrafish as a model system to study cardiovascular development. Trends Cardiovasc. Med..

[21] Bakkers J. (2011). Zebrafish as a model to study cardiac development and human cardiac disease. Cardiovasc. Res..

[22] Langheinrich U., Vacun G., Wagner T. (2003). Zebrafish embryos express an orthologue of HERG and are sensitive toward a range of QT-prolonging drugs inducing severe arrhythmia. Toxicol. Appl. Pharmacol..

[23] Milan D.J., Peterson T.A., Ruskin J.N., Peterson R.T., MacRae C.A. (2003). Drugs that induce repolarization abnormalities cause bradycardia in zebrafish. Circulation.

[24] Teixidó E., Riera-Colomer C., Raldúa D., Pubill D., Escubedo E., Barenys M., López-Arnau R. (2023). First-generation synthetic cathinones produce arrhythmia in zebrafish eleutheroembryos: A new approach methodology for new psychoactive substances cardiotoxicity evaluation. Int. J. Mol. Sci..

[25] Milan D.J., MacRae C.A. (2008). Zebrafish genetic models for arrhythmia. Prog. Biophys. Mol. Biol..

[26] Pazos M.D., Berzosa X., Camarasa J., Pubill D., Escubedo E., Nadal-Gratacós N., López-Arnau R. (2026). Psychostimulant and addictive effects of α-PiHP and MDPiHP, two novel second-generation synthetic cathinones. Neurosci. Appl..

[27] Haverinen J., Hassinen M., Korajoki H., Vornanen M. (2018). Cardiac voltage-gated sodium channel expression and electrophysiological characterization of the sodium current in the zebrafish (*Danio rerio*) ventricle. Prog. Biophys. Mol. Biol..

[28] Bowley G., Kugler E., Wilkinson R., Lawrie A., Van Eeden F., Chico T.J.A., Evans P.C., Noël E.S., Serbanovic-Canic J. (2022). Zebrafish as a tractable model of human cardiovascular disease. Br. J. Pharmacol..

[29] Gauvrit S., Bossaer J., Lee J., Collins M.M. (2022). Modeling human cardiac arrhythmias: Insights from zebrafish. J. Cardiovasc. Dev. Dis..

[30] Vornanen M., Badr A., Haverinen J. (2024). Cardiac arrhythmias in fish induced by natural and anthropogenic changes in environmental conditions. J. Exp. Biol..

[31] Zakaria Z.Z., Benslimane F.M., Nasrallah G.K., Shurbaji S., Younes N.N., Mraiche F., Da’As S.I., Yalcin H.C. (2018). Using zebrafish for investigating the molecular mechanisms of drug-Induced cardiotoxicity. BioMed Res. Int..

[32] Maciag M., Wnorowski A., Bednarz K., Plazinska A. (2022). Evaluation of β-adrenergic ligands for development of pharmacological heart failure and transparency models in zebrafish. Toxicol. Appl. Pharmacol..

[33] Fink M., Callol-Massot C., Chu A., Ruiz-Lozano P., Belmonte J.C.I., Giles W., Bodmer R., Ocorr K. (2009). A new method for detection and quantification of heartbeat parameters in Drosophila, zebrafish, and embryonic mouse hearts. Biotechniques.

[34] Glennon R.A., Young R. (2016). Neurobiology of 3,4-methylenedioxypyrovalerone (MDPV) and α-pyrrolidinovalerophenone (α-PVP). Brain Res. Bull..

[35] Adamowicz P., Jurczyk A., Gil D., Szustowski S. (2020). Case Report A case of intoxication with a new cathinone derivative α -PiHP—A presentation of concentrations in biological specimens. Leg. Med..

[36] Mitchell C.A., Dasgupta S., Zhang S., Stapleton H.M., Volz D.C. (2018). Disruption of nuclear receptor signaling alters triphenyl phosphate-induced cardiotoxicity in zebrafish embryos. Toxicol. Sci..

[37] Yang M., Luan J., Xu Y., Zhao C., Sun M., Feng X. (2022). Cardiotoxicity of zebrafish Induced by 6-benzylaminopurine exposure and its mechanism. Int. J. Mol. Sci..

[38] Reina C., Cardella C., Lo Pinto M., Pucci G., Acuto S., Maggio A., Cavalieri V. (2023). Antioxidant, pro-survival and pro-regenerative effects of conditioned medium from Wharton’s jelly mesenchymal stem cells on developing zebrafish embryos. Int. J. Mol. Sci..

[39] Pucci G., Savoca G., Iacoviello G., Russo G., Forte G.I., Cavalieri V. (2024). Curcumin’s radioprotective effects on zebrafish embryos. Antioxidants.

[40] Guo Z., Ai N., Wang X., Chong C.-M., Ge W., Xu Q. (2025). Multifunctional robotic micromanipulation system for automated cardiovascular disease therapy using zebrafish. npj Biomed. Innov..

[41] Farr G.H., Reid W., Hasegawa E.H., Azzam A., Young I., Li M.L., Olson A.K., Beier D.R., Maves L. (2025). A systems genetics approach identifies roles for proteasome factors in heart development and congenital heart defects. PLoS Genet..

[42] Tamargo J., Le Heuzey J.-Y., Mabo P. (2015). Narrow therapeutic index drugs: A clinical pharmacological consideration to flecainide. Eur. J. Clin. Pharmacol..

[43] Meltzer P.C., Butler D., Deschamps J.R., Madras B.K. (2006). 1-(4-Methylphenyl)-2-pyrrolidin-1-yl-pentan-1-one (Pyrovalerone) Analogues:  A Promising Class of Monoamine Uptake Inhibitors. J. Med. Chem..

