# Supplementary material for: Integrated Assessment of Neurobehavioral and Cardiotoxic Effects of Pyrrolidine-Containing Cathinones in Zebrafish: Structural Determinants of Functional Safety Profiles
Source: Int J Mol Sci. 2026 Mar 30;27(7):3141. doi: 10.3390/ijms27073141 (PMC13073605; doi:10.3390/ijms27073141)
Supplement: Supplementary file 1 [file ijms-27-03141-s001.zip › ijms-4195719-supplementary.pdf]

Supplementary Information

Integrated Assessment of Neurobehavioral and Cardiotoxic Effects of Pyrrolidine-Containing Cathinones in Zebrafish: Structural Determinants of Functional Safety Profiles

Ouwais Aljabasini, Niki Tagkalidou, Martalu D. Pazos, Guillermo García-Díez, Eva Prats, Roger Seco, Xavier Berzosa, Raul Lopez-Arnau, and Demetrio Raldúa

Supplementary Methods

1. Synthesis and characterization of the pyrrolidinophenone derivatives

1.1. Synthesis

The synthesis of the different cathinones was performed through three sequential steps, following the procedure described by Meltzer et al. (2006) [43]. The overall route of synthesis is shown in the following figure.

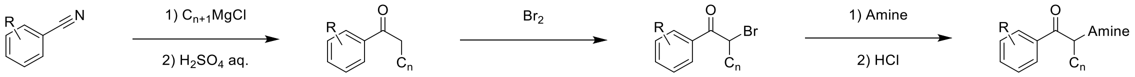

In general, the substituted benzonitrile is reacted with the appropriate Grignard reagent to obtain the corresponding ketone, after an acidic treatment. Afterwards, the ketone is brominated in the α position. Finally, the bromoketone is reacted with the corresponding amine to obtain the desired cathinone as a free base. The hydrochloride salt can be produced by reaction with hydrogen chloride.

1.2. Characterization

A Varian 400-MR spectrometer has been used to record 1H-NMR and 13C-NMR spectra. Chemical shifts are reported in part per million (ppm) on the δ scale. Coupling constants are reported in Hertz (Hz).

4-F-3-Me-α-PVP

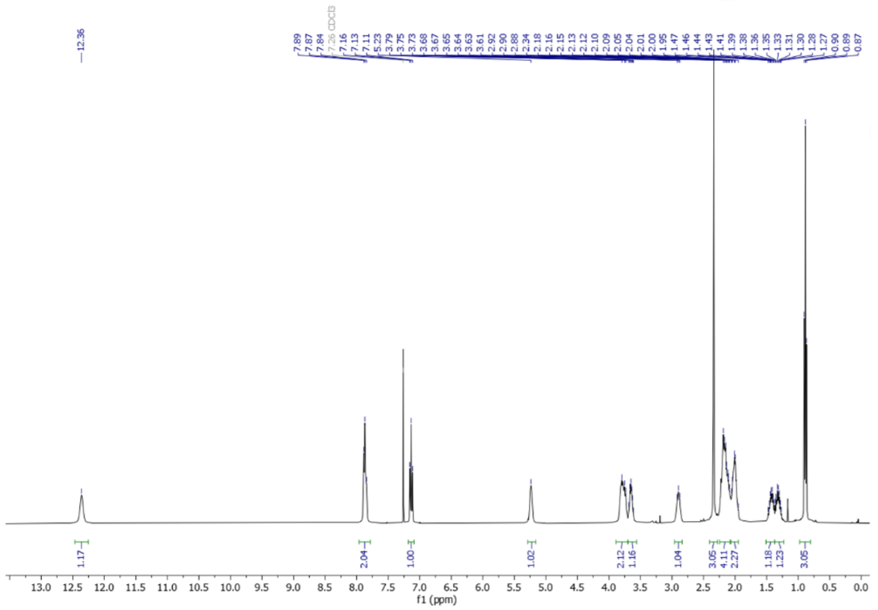

^{1}H NMR (400 MHz, $CDCl_3$) δ 12.36 (s, 1H), 7.89 – 7.84 (m, 2H), 7.13 (t, *J* = 8.7 Hz, 1H), 5.23 (s, 1H), 3.89 – 3.70 (m, 2H), 3.64 (dd, *J* = 11.1, 5.5 Hz, 1H), 2.90 (t, *J* = 8.7 Hz, 1H), 2.34 (s, 3H), 2.24 – 2.08 (m, 4H), 2.06 – 1.95 (m, 2H), 1.51 – 1.37 (m, 1H), 1.32 (td, *J* = 12.6, 6.1 Hz, 1H), 0.89 (t, *J* = 7.2 Hz, 3H).

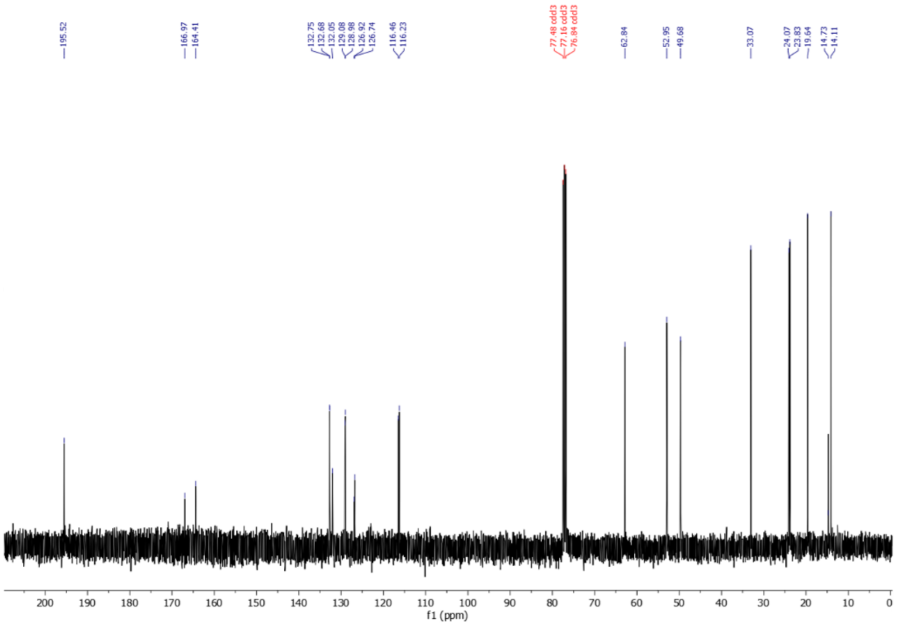

^{13}C NMR (100 MHz, $CDCl_3$) δ 195.52, 166.97-164.41, 132.75-132.68, 132.05, 129.08-128.98, 126.92-126.74, 116.46-116.23, 62.84, 52.95, 49.68, 33.07, 24.07, 23.83, 19.64, 14.73, 14.11.

α-D2PV

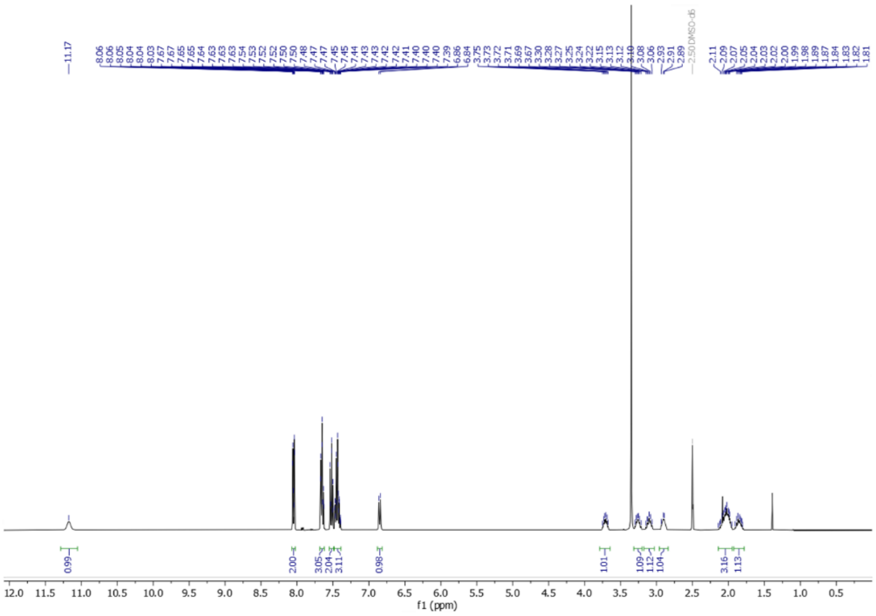

^{1}H NMR (400 MHz, DMSO-d$_6$) δ 11.17 (s, 1H), 8.07 – 8.02 (m, 2H), 7.65 (td, *J* = 7.8, 1.7 Hz, 3H), 7.55 – 7.49 (m, 2H), 7.48 – 7.39 (m, 3H), 6.85 (d, *J* = 8.9 Hz, 1H), 3.71 (h, *J* = 6.3 Hz, 1H), 3.32 – 3.20 (m, 1H), 3.17 – 3.03 (m, 1H), 2.96 – 2.84 (m, 1H), 2.14 – 1.95 (m, 3H), 1.93 – 1.78 (m, 1H).

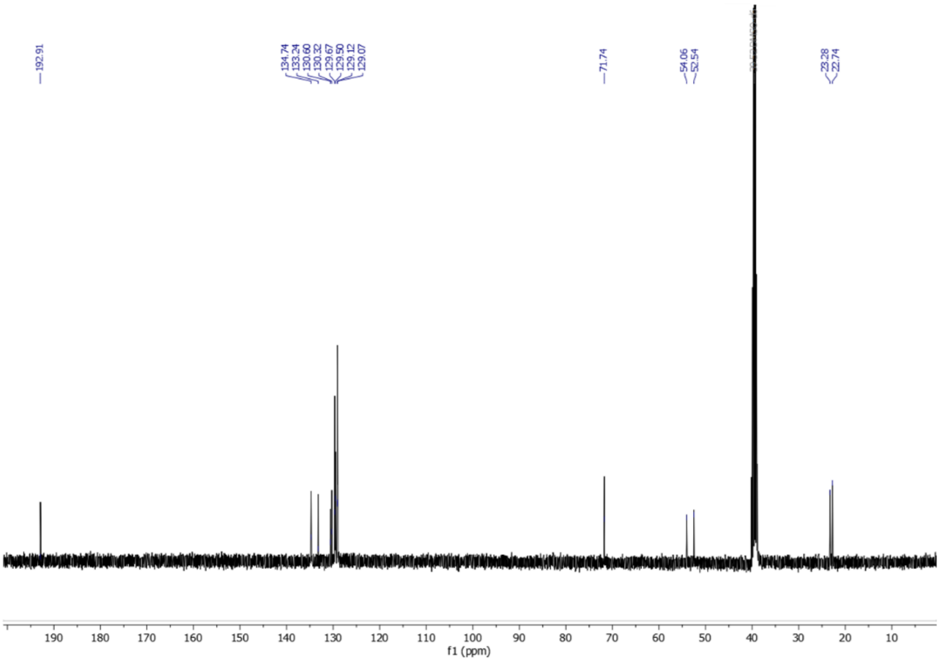

^{13}C NMR (100 MHz, DMSO-d$_6$) δ 192.91, 134.74, 133.24, 130.60, 130.32, 129.67, 129.50, 129.12, 129.07, 71.74, 54.06, 52.54, 23.28, 22.74.

3-Cl-α-PVP

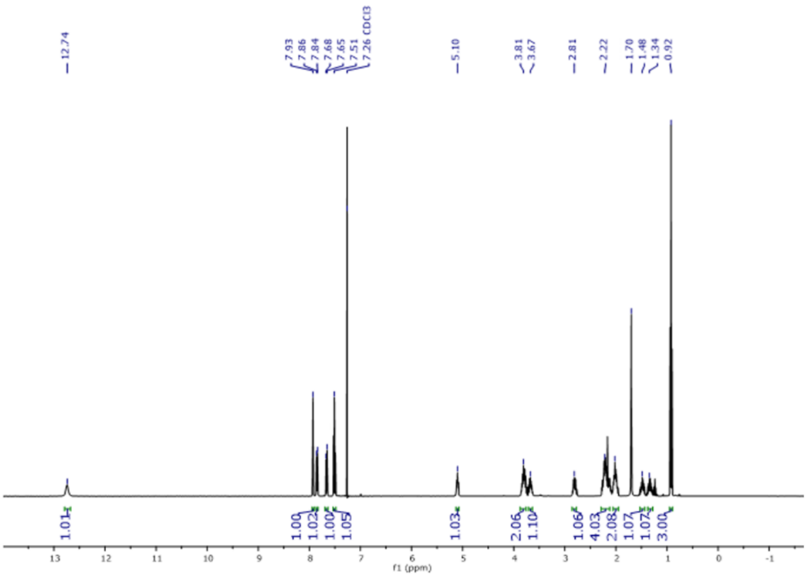

^{1}H NMR (400 MHz, $CDCl_3$) δ 12.74 (s, 1H), 7.93 (t, *J* = 1.8 Hz, 1H), 7.85 (dt, *J* = 7.8, 1.3 Hz, 1H), 7.67 (ddd, *J* = 8.0, 2.1, 0.9 Hz, 1H), 7.51 (t, *J* = 7.8 Hz, 1H), 5.10 (m, 1H), 3.81 (m, 2H), 3.67 (m, 1H), 2.81 (m, 1H), 2.22 (m, 4H), 2.02 (m, 2H), 1.48 (m, 1H), 1.48 (m, 1H), 0.92 (t, *J* = 7.3 Hz, 3H).

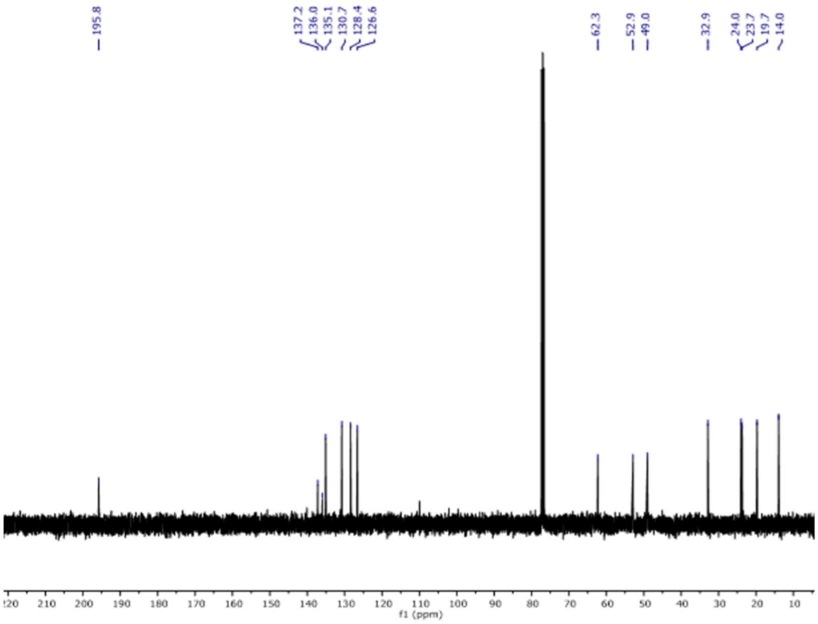

^{13}C NMR (100 MHz, $CDCl_3$) δ 195.8, 137.2, 136.0, 135.1, 130.7, 130.0, 128.4, 62.3, 52.9, 49.0, 32.8, 23.9, 23.7, 19.7, 13.9.

4-Cl-α-PVP

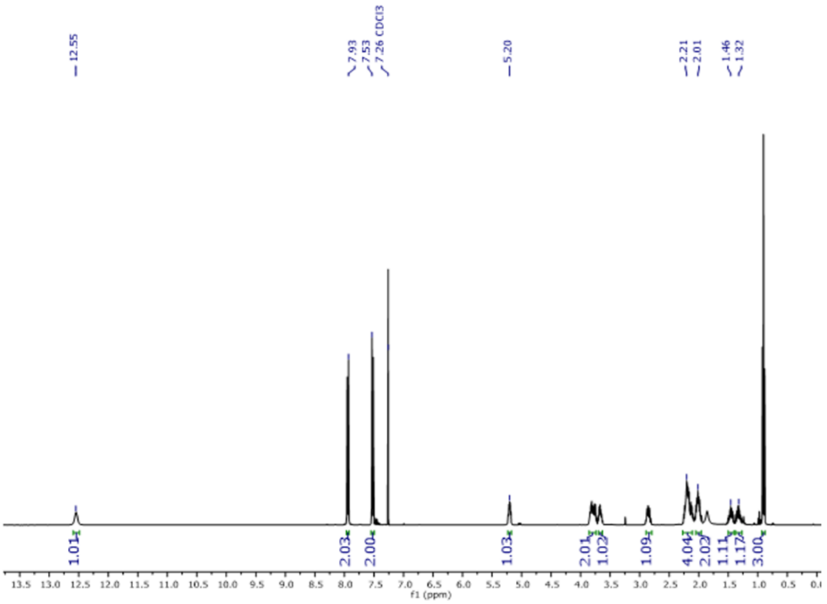

^{1}H NMR (400 MHz, $CDCl_3$) δ 12.55 (s, 1H), 7.94 (dt, *J* = 7.8, 1.3 Hz, 2H), 7.52 (dt, *J* = 7.8, 1.3 Hz, 1H), 5.20 (dt, *J* = 9.0, 5.1 Hz 1H), 3.81 (m, 2H), 3.66 (m, 1H), 2.81 (m, 1H), 2.21 (m, 4H), 2.01 (m, 2H), 1.46 (m, 1H), 1.32 (m, 1H), 0.90 (t, *J* = 7.3 Hz, 3H).

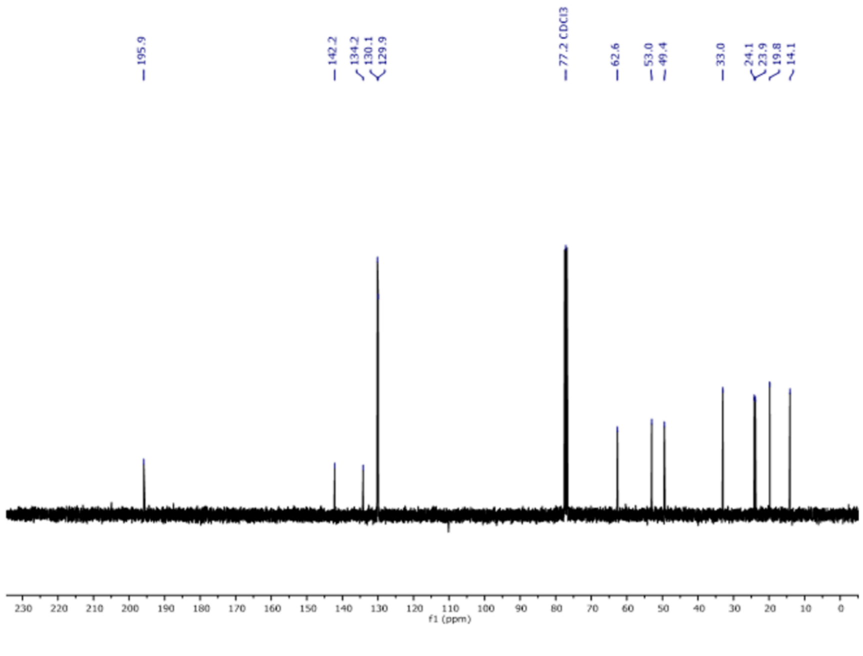

^{13}C NMR (100 MHz, $CDCl_3$) δ 195.9, 142.2, 134.2, 130.1, 129.9, 62.6, 53.0, 49.4, 33.0, 24.1, 23.9, 19.8, 14.1.

3,4-Cl-α-PVP

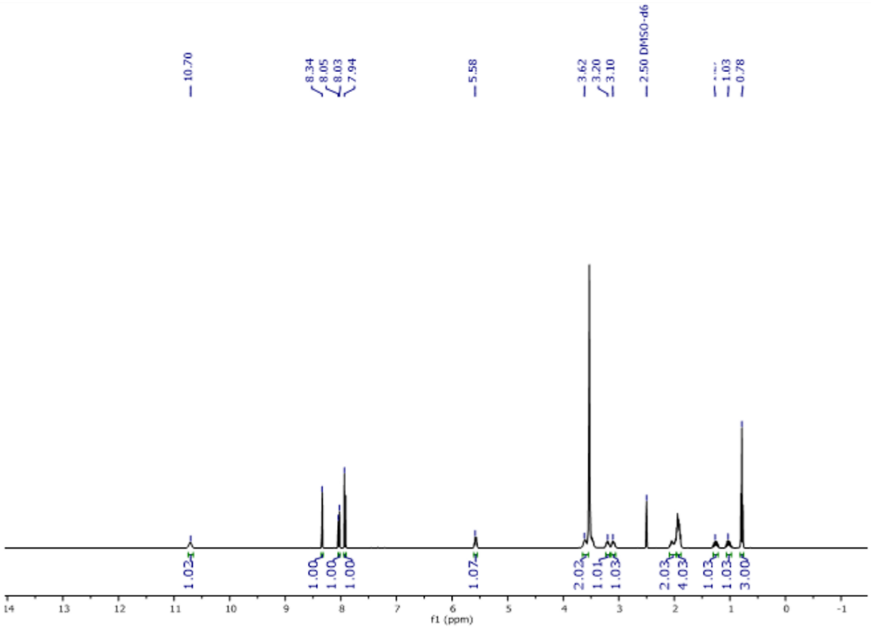

^{1}H NMR (400 MHz, $CDCl_3$) δ 10.70 (s, 1H), 8.34 (dd, *J* = 2.0, 0.6 Hz, 1H), 8.03 (dd, *J* = 8.5, 2.1 Hz, 1H), 7.93 (d, *J* = 8.4 Hz, 1H), 5.57 (dt, *J* = 8.5, 5.6 Hz, 1H), 3.62 (m, 2H), 3.20 (m, 1H), 3.10 (m, 1H), 2.03 (m, 2H), 1.92 (m, 4H), 1.27 (m, 1H), 1.03 (m, 1H), 0.78 (t, *J* = 7.3 Hz, 3H).

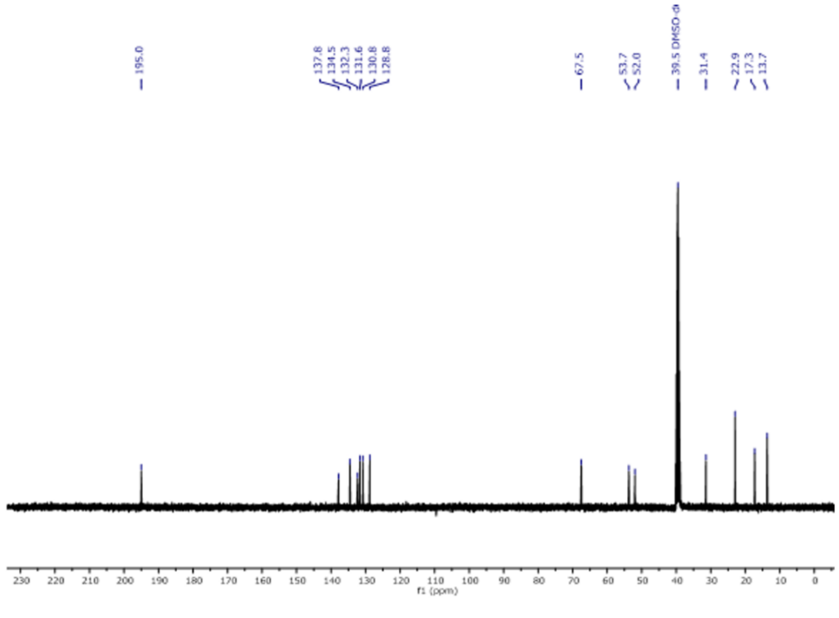

^{13}C NMR (100 MHz, $CDCl_3$) δ 195.0, 137.8, 134.5, 132.3, 131.6, 130.8, 128.8, 67.5, 53.7, 52.0, 31.4, 22.9, 17.3, 13.7.

MDPV

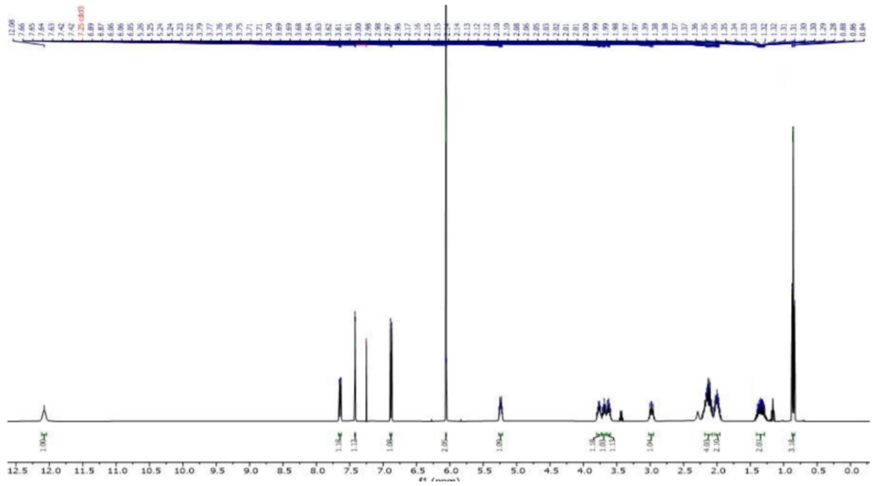

^{1}H NMR (400 MHz, $CDCl_3$) δ 12.08 (s, 1H), 7.64 (dd, *J* = 8.3, 1.8 Hz, 1H), 7.42 (d, *J* = 1.7 Hz, 1H), 6.88 (d, *J* = 8.2 Hz, 1H), 6.06 (s, 2H), 5.24 (dt, *J* = 8.2, 5.0 Hz, 1H), 3.81 – 3.73 (m, 1H), 3.73 – 3.65 (m, 1H), 3.65 – 3.58 (m, 1H), 3.04 – 2.92 (m, 1H), 2.22 – 2.06 (m, 4H), 2.00 (ddt, *J* = 13.5, 10.6, 5.3 Hz, 2H), 1.43 – 1.26 (m, 2H), 0.86 (t, *J* = 7.3 Hz, 3H).

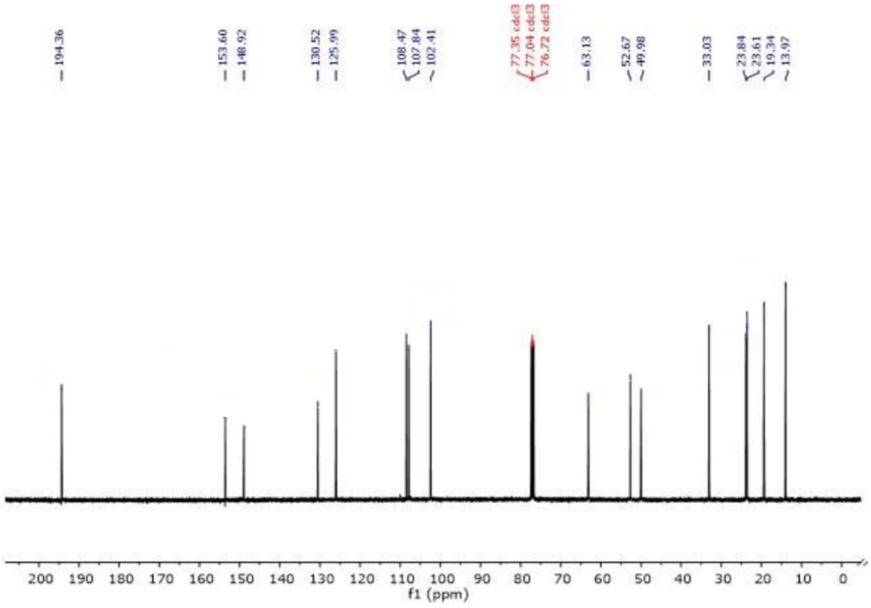

^{13}C NMR (100 MHz, $CDCl_3$) δ 194.38, 153.61, 148.94, 130.53, 126.00, 108.48, 107.85, 102.42, 77.36, 77.25, 77.04, 76.73, 63.13, 52.67, 49.99, 33.03, 23.85, 23.61, 19.34, 13.97.

MDPiHP

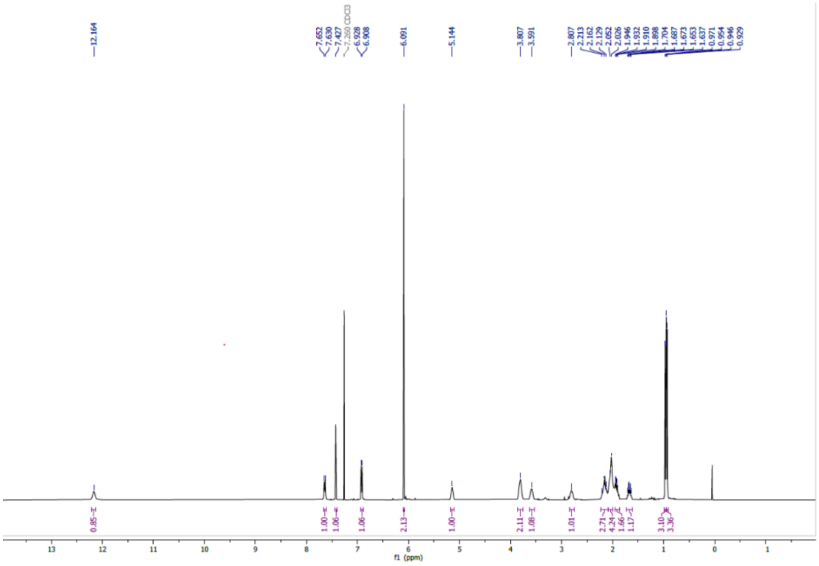

^{1}H NMR (400 MHz, $CDCl_3$) δ 12.16 (s, 1H), 7.64 (d, *J* = 8.9 Hz, 1H), 7.43 (s, 1H), 6.92 (d, *J* = 8.1 Hz, 1H), 6.09 (s, 2H), 5.14 (s, 1H), 3.81 (s, 2H), 3.59 (s, 1H), 2.81 (s, 1H), 2.23 – 2.08 (m, 3H), 2.04 (d, *J* = 10.7 Hz, 4H), 1.92 (dd, *J* = 13.8, 5.4 Hz, 1H), 1.73 – 1.61 (m, 1H), 0.96 (d, *J* = 6.6 Hz, 3H), 0.94 (d, *J* = 6.5 Hz, 3H).

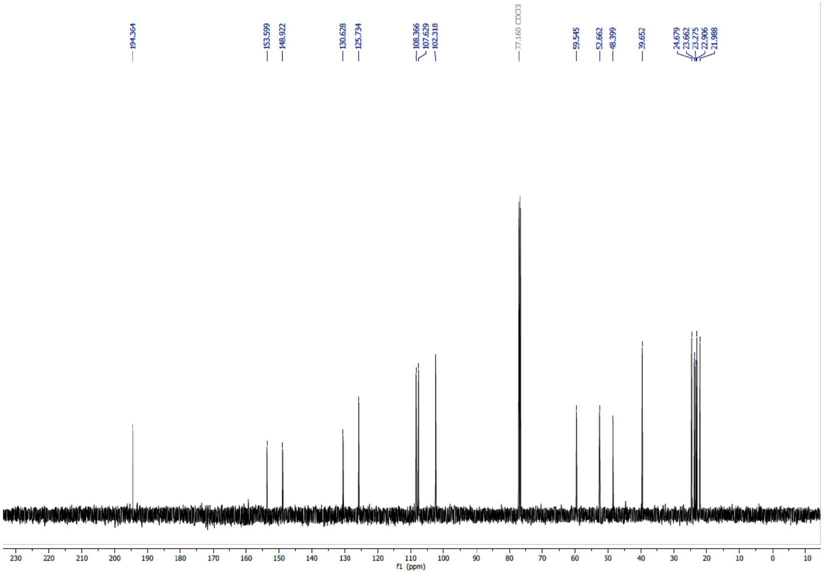

^{13}C NMR (100 MHz, $CDCl_3$) δ 194.36, 153.60, 148.92, 130.63, 125.73, 108.37, 107.63, 102.32, 59.54, 52.66, 48.40, 39.65, 24.68 65, 23.66 65, 23.2765, 22.91, 21.99.

α-PVP

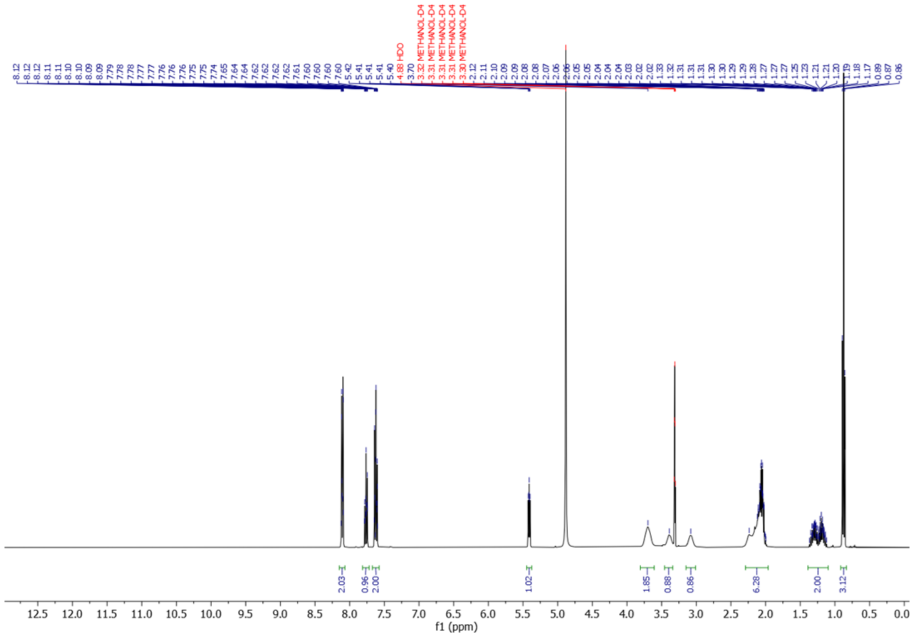

^{1}H NMR (400 MHz, CD_3OD) δ 8.15 – 8.07 (m, 2H), 7.81 – 7.72 (m, 1H), 7.67 – 7.58 (m, 2H), 5.45 – 5.37 (m, 1H), 3.70 (s, 2H), 3.39 (s, 1H), 3.08 (s, 1H), 2.29 – 1.96 (m, 6H), 1.39 – 1.10 (m, 2H), 0.87 (t, *J* = 7.3 Hz, 3H).

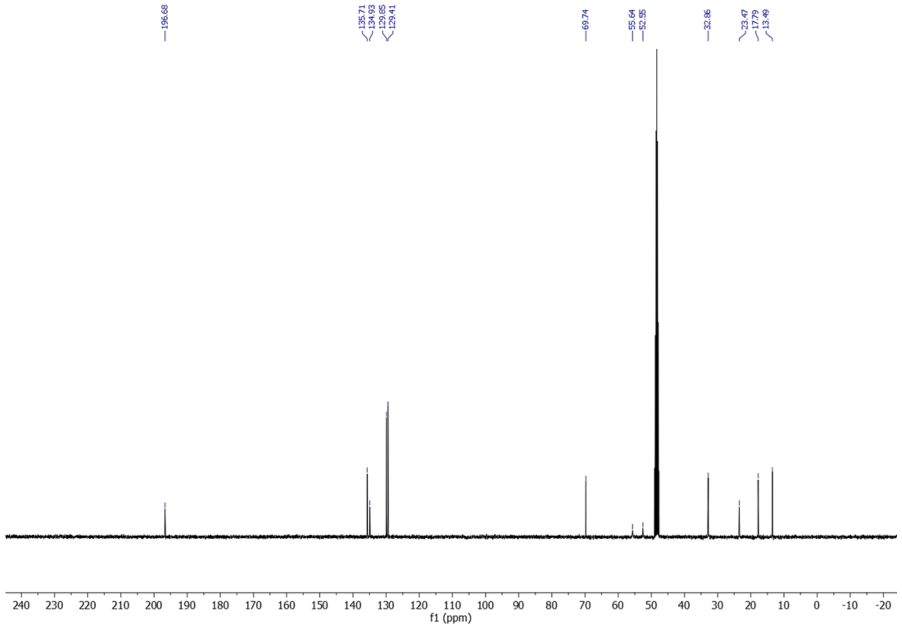

^{13}C NMR (101 MHz, CD_3OD) δ 196.68, 135.71, 134.93, 129.85, 129.41, 69.74, 55.64, 52.55, 32.86, 23.47, 17.79, 13.49.

α-PiHP

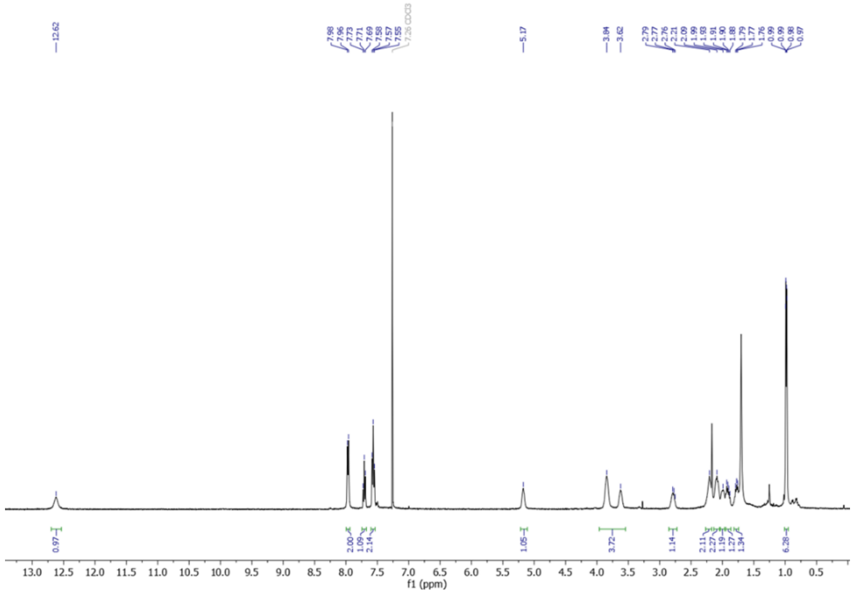

^{1}H NMR (400 MHz, $CDCl_3$) δ 12.62 (s, 1H), 7.97 (d, *J* = 7.7 Hz, 2H), 7.71 (t, *J* = 7.4 Hz, 1H), 7.57 (t, *J* = 7.5 Hz, 2H), 5.17 (s, 1H), 3.73 (d, *J* = 88.3 Hz, 3H), 2.78 (d, *J* = 8.4 Hz, 1H), 2.21 (s, 2H), 2.09 (s, 2H), 1.99 (s, 1H), 1.91 (dd, *J* = 13.4, 6.9 Hz, 1H), 1.82 – 1.74 (m, 1H), 0.98 (dd, *J* = 6.5, 2.0 Hz, 6H).

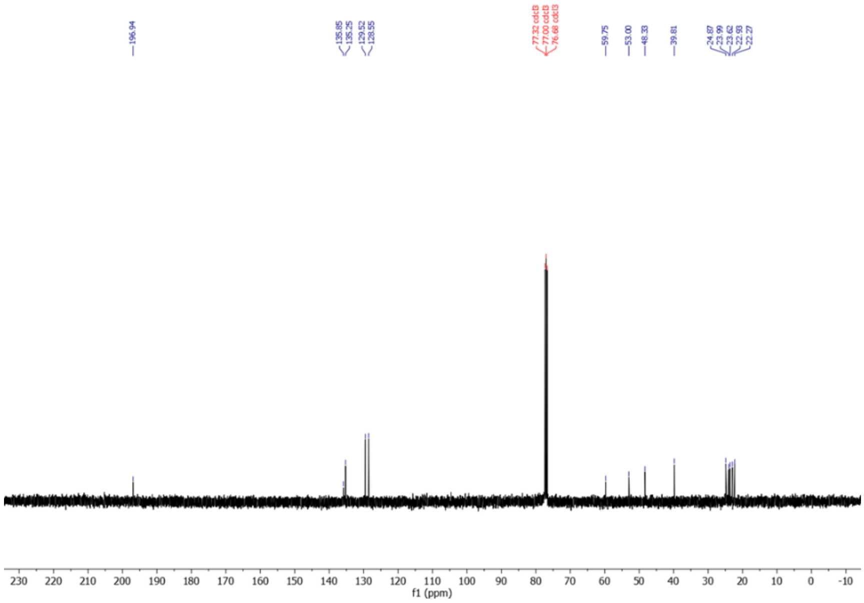

^{13}C NMR (100 MHz, $CDCl_3$) δ 196.94, 135.85, 135.25, 129.52, 128.55, 59.75, 53.00, 48.33, 39.81, 24.87, 23.99, 23.62, 22.93, 22.27.

2. Locomotor activity analysis

Locomotor activity was quantified as basal locomotor activity (BLA) using dynamic pixel analysis. Measurements were summarized in 15-min bins (0–15 to 105–120 min). Data were normalized within each independent experiment and time bin to the median of time-matched vehicle controls (control median = 100), enabling pooling across batches. Outliers were removed per compound × concentration × time bin using the 1.5×IQR criterion. Late-phase locomotor potency was summarized at 105–120 min and expressed as an EC50-like metric estimated by log-linear interpolation between the two concentrations (among 50 nM, 500 nM and 5 µM) bracketing 50% residual activity (50% of control). If 50% inhibition was not reached at 5 µM, EC50-like was treated as right-censored (>5 µM).

Supplementary Tables

Table S1. Late-phase (105–120 min) descriptive statistics for normalized BLA by compound and concentration.

Compound	Condition	Dose (µM)	Median [IQR]	n (incl/total)
MDPV	Control	0	96.9 [62.1–126.4]	51/54
	50 nM	0.05	72.6 [45.0–141.3]	34/34
	500 nM	0.5	44.2 [16.7–83.1]	53/54
	5 µM	5	17.3 [8.8–30.5]	51/54
MDPiHP	Control	0	89.5 [36.3–112.3]	31/38
	50 nM	0.05	89.5 [50.1–190.8]	34/38
	500 nM	0.5	58.4 [23.8–184.7]	38/38
	5 µM	5	33.5 [14.9–121.6]	37/38
α-PVP	Control	0	83.6 [32.4–141.5]	32/38
	50 nM	0.05	52.1 [26.9–77.2]	33/38
	500 nM	0.5	39.8 [20.5–62.5]	32/38
	5 µM	5	30.4 [9.1–49.1]	34/38
α-D2PV	Control	0	100.0 [27.5–134.0]	38/38
	50 nM	0.05	64.1 [26.1–107.9]	37/38
	500 nM	0.5	80.2 [35.0–144.5]	38/38
	5 µM	5	43.5 [19.8–71.6]	37/38
α-PiHP	Control	0	87.5 [63.2–138.4]	32/38
	50 nM	0.05	141.0 [83.4–193.0]	37/38
	500 nM	0.5	78.9 [42.4–163.5]	38/38
	5 µM	5	32.1 [15.9–47.1]	33/38
3-Cl-α-PVP	Control	0	100.0 [45.5–170.8]	37/38
	50 nM	0.05	56.9 [35.4–145.9]	36/38
	500 nM	0.5	45.6 [21.8–65.7]	36/38
	5 µM	5	38.9 [24.1–53.8]	36/38
4-Cl-α-PVP	Control	0	97.8 [46.0–223.6]	35/38
	50 nM	0.05	59.3 [26.8–141.5]	37/38

	500 nM	0.5	39.3 [26.1–79.0]	36/38
	5 µM	5	14.3 [5.8–34.0]	34/38
3,4-Cl-α-PVP	Control	0	81.6 [38.6–126.3]	31/38
	50 nM	0.05	127.0 [42.1–281.4]	37/38
	500 nM	0.5	111.7 [60.0–163.2]	35/38
	5 µM	5	11.7 [2.6–25.8]	35/38
4-F-3-Me-α-PVP	Control	0	101.6 [47.4–206.0]	38/38
	50 nM	0.05	240.4 [94.2–340.6]	36/38
	500 nM	0.5	116.5 [61.5–163.7]	36/38
	5 µM	5	84.9 [16.5–192.2]	38/38

Values are median [IQR] of pooled replicate-level normalized BLA after outlier removal (1.5×IQR). n indicates included/total observations

Table S2. Locomotor EC_{50}-like summary derived from late-phase medians (105–120 min)

Compound	Median 50nM	Median 500nM	Median 5µM	EC_{50}-like 5 µM	Right-censored (>5µM)
MDPV	72.633	44.197	17.318	0.313	FALSE
MDPiHP	89.494	58.408	33.494	1.088	FALSE
α-PVP	52.141	39.775	30.398	0.074	FALSE
α-D2PV	64.053	80.224	43.523	3.330	FALSE
α-PiHP	141.031	78.870	32.056	2.069	FALSE
3-Cl-α-PVP	56.907	45.631	38.919	0.205	FALSE
4-Cl-α-PVP	59.308	39.300	14.326	0.146	FALSE
3,4-Cl-α-PVP	127.042	111.691	11.650	2.068	FALSE
4-F-3-Me-α-PVP	240.370	116.468	84.889	>5	TRUE

EC_{50}-like values were estimated by log-linear interpolation between concentrations bracketing 50% residual activity. If 50% inhibition was not reached at 5 µM, EC_{50}-like is reported as >5 µM.

Table S3. Complete nonparametric statistics for locomotion at representative windows: Kruskal–Wallis and Dunn's post hoc tests (Bonferroni-adjusted) versus control.

Compound	Window (min)	*H*	*p*	Dunn *p* (50 nM)	Dunn *p* (500 nM)	Dunn *p* (5 µM)
MDPV	0-15	113.87	<0.001	1.000	0.050	<0.001
	45-60	65.12	<0.001	1.000	1.000	<0.001
	105-120	66.42	<0.001	1.000	0.001	<0.001
MDPiHP	0-15	97.01	<0.001	0.999	0.020	<0.001
	45-60	45.62	<0.001	0.042	1.000	<0.001
	105-120	8.76	0.033	0.706	1.000	0.317
α-PVP	0-15	73.55	<0.001	0.764	0.010	<0.001
	45-60	40.70	<0.001	1.000	0.108	<0.001
	105-120	15.65	0.001	0.330	0.056	<0.001
α-D2PV	0-15	77.49	<0.001	1.000	0.010	<0.001
	45-60	45.05	<0.001	0.407	0.089	<0.001
	105-120	9.43	0.024	1.000	1.000	0.019
α-PiHP	0-15	110.26	<0.001	1.000	<0.001	<0.001
	45-60	72.32	<0.001	0.037	<0.001	<0.001
	105-120	39.46	<0.001	0.295	1.000	<0.001
3-Cl-α-PVP	0-15	90.25	<0.001	1.000	<0.001	<0.001
	45-60	49.54	<0.001	<0.001	<0.001	<0.001
	105-120	26.54	<0.001	0.252	<0.001	<0.001
4-Cl-α-PVP	0-15	97.42	<0.001	0.475	<0.001	<0.001
	45-60	61.68	<0.001	0.041	0.004	<0.001
	105-120	38.75	<0.001	0.232	0.010	<0.001
3,4-Cl-α-PVP	0-15	110.93	<0.001	0.288	<0.001	<0.001
	45-60	66.19	<0.001	0.268	0.070	<0.001
	105-120	59.55	<0.001	0.226	0.718	<0.001
4-F-3-Me-α-PVP	0-15	101.69	<0.001	0.166	<0.001	<0.001
	45-60	81.10	<0.001	0.603	<0.001	<0.001
	105-120	11.40	0.010	0.064	1.000	0.970

Supplementary Figures

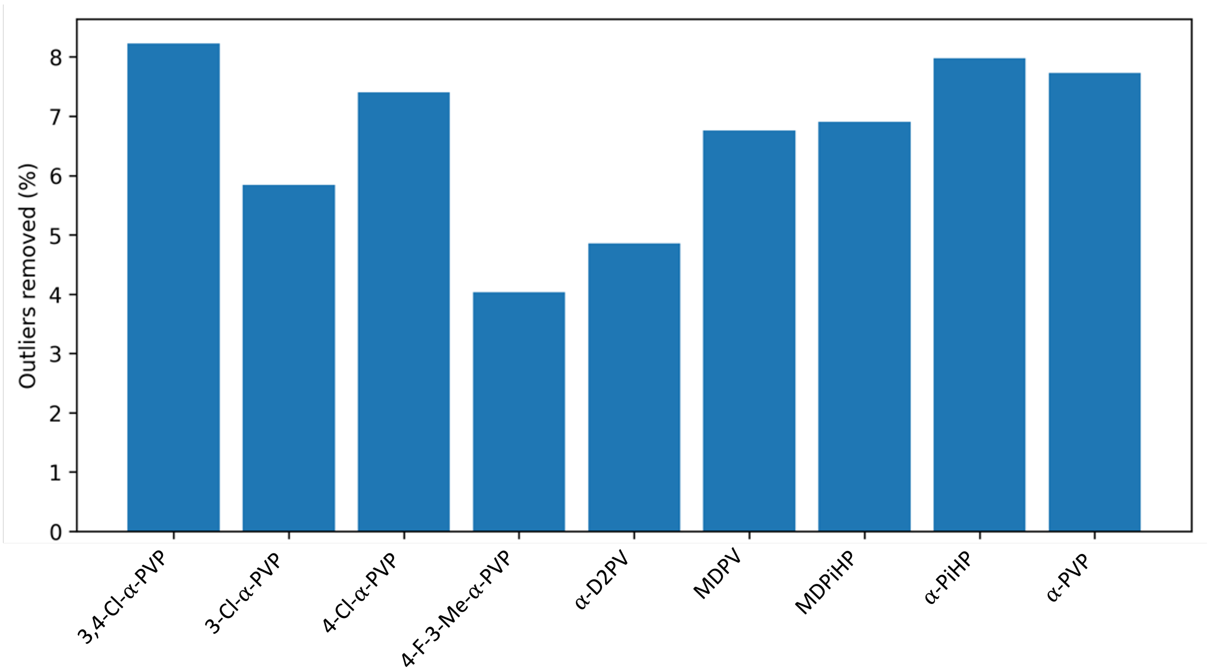

Figure S1. Summary of outlier removal rates across compounds (percentage of replicate-level observations flagged by the 1.5×IQR criterion).

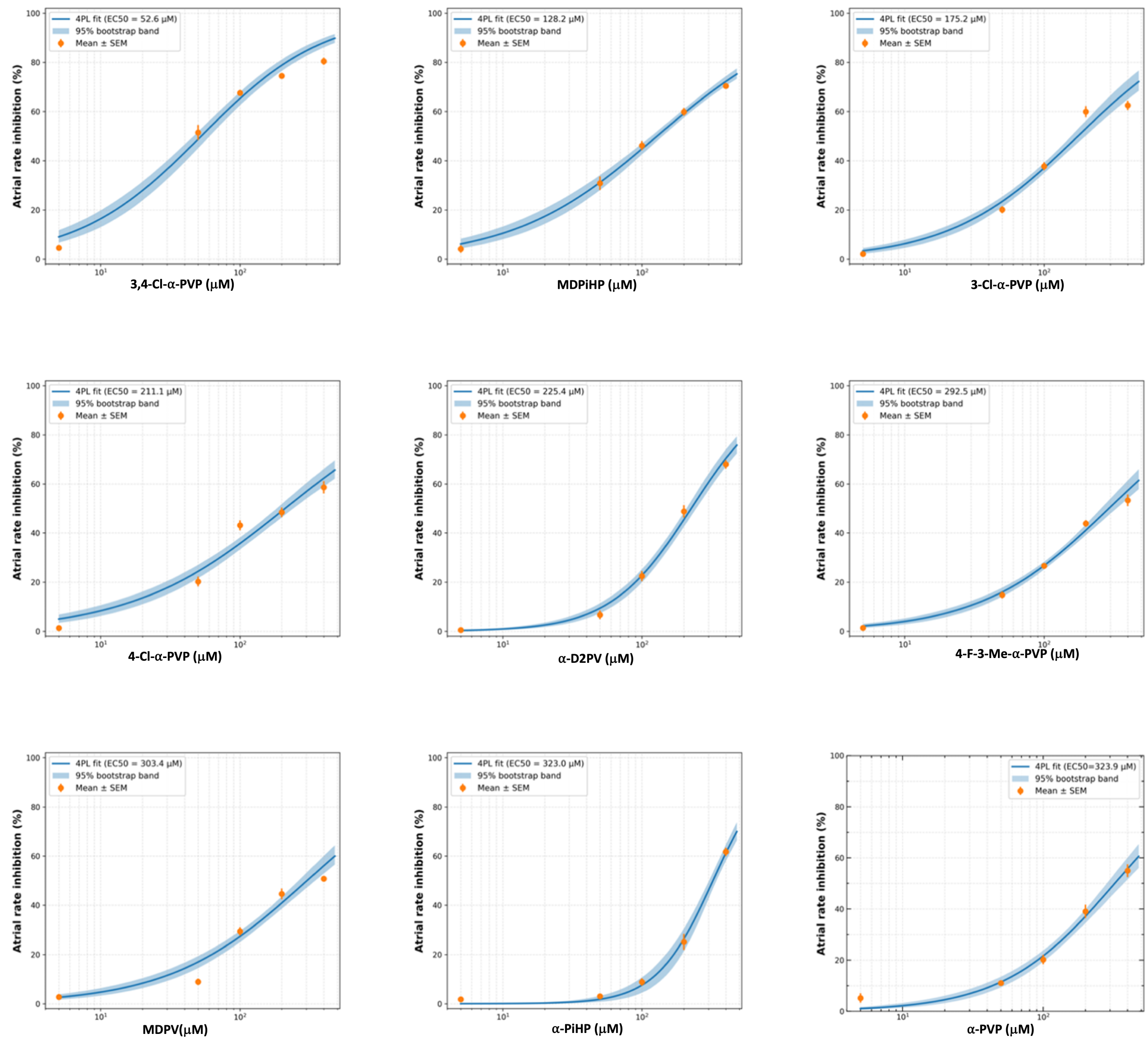

Supplementary Figure S2. Individual concentration–response curves for atrial chronotropy. Concentration–response relationships for atrial rate inhibition induced by individual pyrrolidinophenone derivatives in zebrafish embryos. Symbols represent mean ± SEM at each concentration. Solid lines indicate 4PL model fits, and shaded areas represent 95% confidence intervals derived from nonparametric bootstrap resampling (n = 2000). Chemicals in the panel are ordered according to increasing atrial chronotropic potency (ascending EC_{50} values).

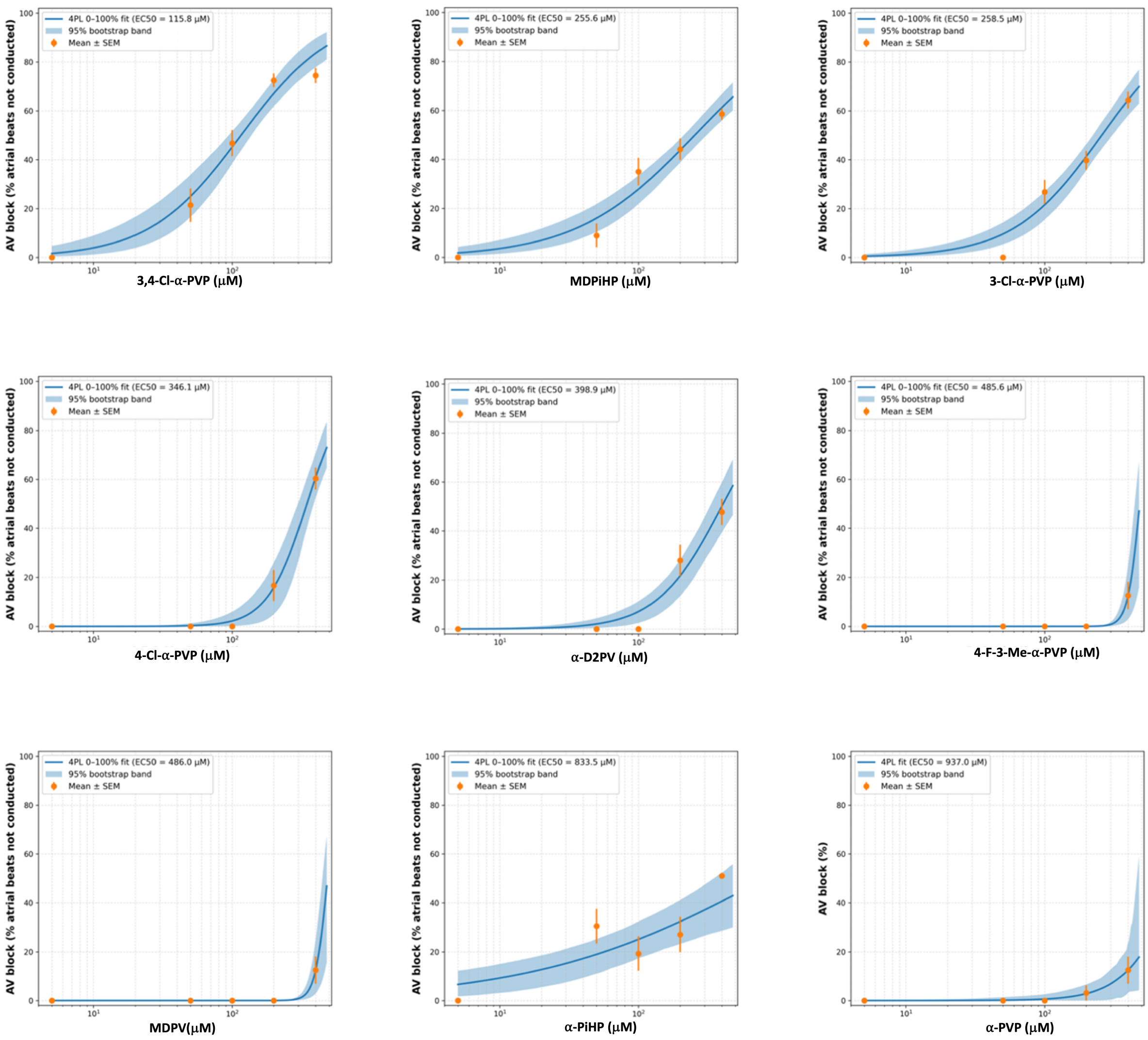

Supplementary Figure S3. Individual concentration–response curves for atrioventricular conduction. Concentration–response relationships for AV block induced by individual pyrrolidinophenone derivatives in zebrafish embryos. Symbols represent mean ± SEM values at each concentration. Solid lines indicate 4PL model fits, and shaded areas represent 95% confidence intervals derived from nonparametric bootstrap resampling (n = 2000). Chemicals in the panel are ordered according to increasing AV-blocking potency (ascending EC_{50} values).

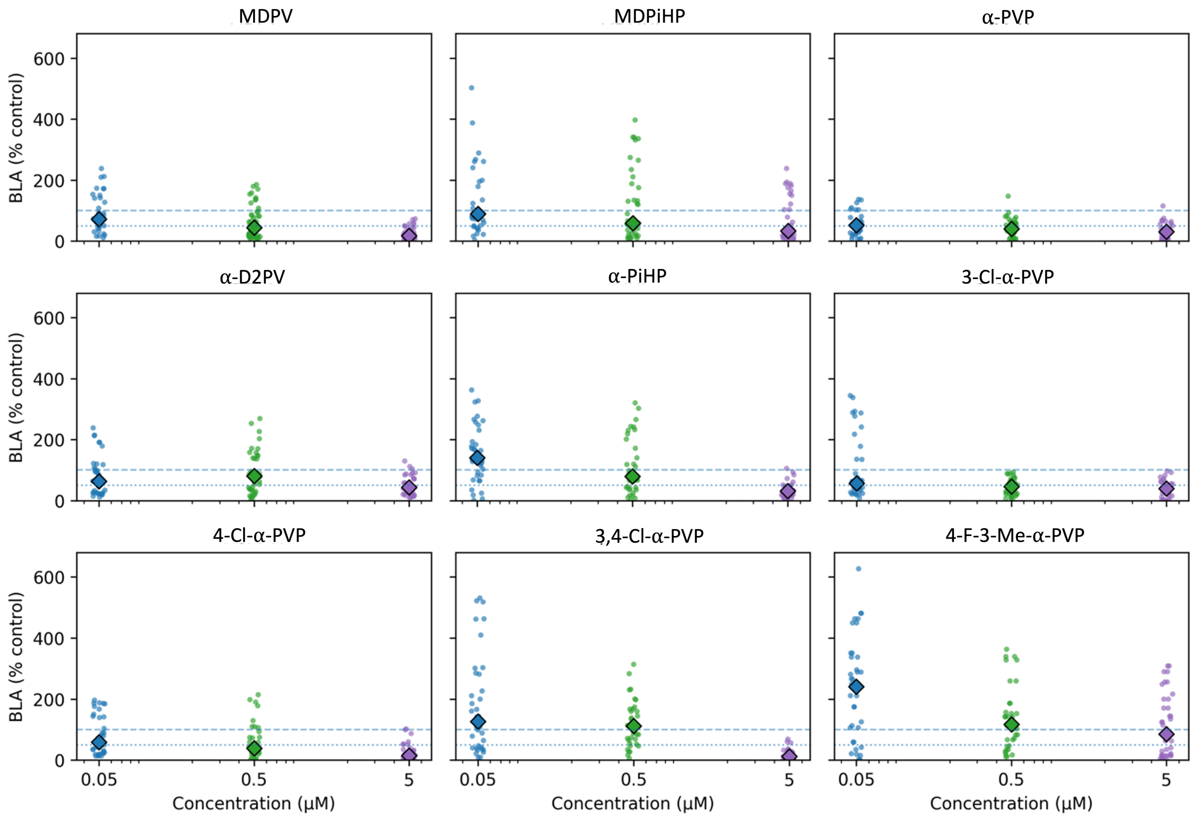

Figure S4. Late-phase (105–120 min) locomotor dose–response profiles. Dots represent replicate-level normalized BLA after outlier removal (1.5×IQR); diamonds indicate medians for each concentration.

Supplementary Datasets

Dataset S1. (1) Cardiac chamber heart rate: Normalized heart rate values at the atrium and ventricle in 3 days post fertilization zebrafish embryos control and treated with the nine chemicals at three different concentrations. (2) Pooled replicate-level locomotor activity data (normalized), including time bin, compound, concentration, and outlier flags.
